# Exploring In Vivo Metal Chelation as an Approach for Pretargeted PET Imaging

**DOI:** 10.1021/acsomega.4c10050

**Published:** 2025-05-07

**Authors:** Aishwarya Mishra, George Keeling, Jana Kim, Rafael T. M. de Rosales

**Affiliations:** School of Biomedical Engineering & Imaging Sciences, 4616 King’s College London, St Thomas’ Hospital, London SE1 7EH, U.K.

## Abstract

Pretargeted PET imaging has emerged as a leading strategy for tracking long-circulating agents such as antibodies and nanoparticle-drug delivery systems with short-lived isotopes. Compared to the conventional direct radiolabeling approach, pretargeting benefits from high sensitivity and spatial resolution of PET while minimizing radiation doses and nonspecific accumulation of radioactivity. In addition, it allows for long-term *in vivo* tracking possibilities. However, a pretargeting approach that can utilize readily available radionuclides as obtained from the generator/cyclotron without the need of complex radiochemical synthesis is highly desirable. Here, we report a metal chelation pretargeting system based on the ^68^Ga chelator tris­(hydroxypyridinone) (THP). THP can be radiolabeled at low concentrations with as-obtained generator-produced radionuclide gallium-68 (*t*_1/2_ = 68 min) at room temperature and physiological conditions with high efficiency. The bifunctional chelator THP-NCS was conjugated to either PEGylated liposomes or a bone-targeting aminobisphosphonate (pamidronate) to examine the metal chelation pretargeted imaging system in both long-circulating nanomedicines and short-circulating small molecules, respectively. *In vivo* imaging experiments were performed in healthy BalB/c mice at multiple time points. For liposomal pretargeting, the fraction of liposomes circulating in the blood was efficiently radiolabeled *in vivo*, but limited *in vivo* radiolabeling was observed for liposomes that had accumulated in the liver and spleen. The pretargeting of the small-molecule, bone-targeting bisphosphonate THP-Pam showed moderate *in vivo* radiolabeling in bones. Overall, based on this study, the metal chelation method appears to allow easy pretargeting for agents present in the blood and bones but with limited success in other organs.

## Graphical abstract

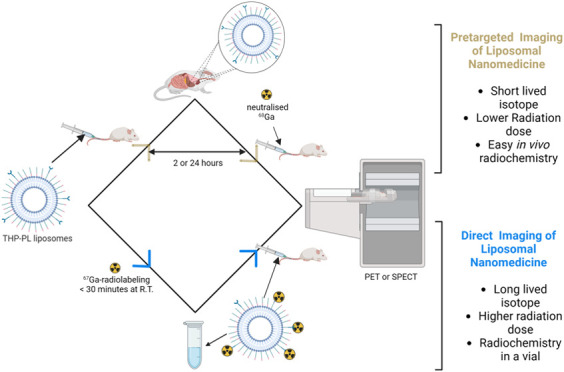

## Introduction

Nuclear imaging of long-circulating macromolecules such as antibodies and liposomal nanomedicines allows in vivo tracking, providing insight into their efficacy and pharmacokinetics. The various radiolabeling approaches available allow labeling of these macromolecules with a vast library of radioisotopes.^1^ However, the slow kinetics of target accumulation of these macromolecules pose a challenge in choosing the half-life of radionuclides that can be used to image them in vivo.^2^ To harness the full potential of nuclear imaging, there is a need to match the blood half-lives of these particles to the radionuclides with multiday half-lives such as ^111^In (*t*_1/2_ = 2.8 d), ^89^Zr (*t*_1/2_ = 3.3 d), ^177^Lu (*t*_1/2_ = 6.7 d), ^186^Re (*t*_1/2_ = 3.7 d), and ^131^I (*t*_1/2_ = 8.0 d), among others. The use of such radionuclides, however, is a double-edged sword. The long-lived therapeutic and diagnostic radionuclides suitable for imaging of these long-circulating macromolecules create significant clinical considerations: low therapeutic doses for radiotherapy to the target tissue, high radiation doses to healthy tissue during circulation, and shielding requirements for patients and caretakers in the clinical setting.

The pretargeted imaging approach can help us overcome these limitations by decoupling the tumor-targeting agent (here, nanomedicines and small molecules) from its radiolabeled tag and perform in vivo labeling.^3–12^ The pretargeted strategy allows longitudinal imaging beyond the half-life of the radionuclide while minimizing the radiation dose to nontarget organs.^13^ Pretargeted imaging has been successfully applied for tracking antibodies with slow pharmacokinetics, similar to nanomedicines, allowing the use of short-lived isotopes with lower radiation doses.^14,15^ These examples include different approaches to pretargeting including bioorthogonal chemistry, bispecific antibody–hapten binding, and avidin–biotin binding. These approaches have been applied to antibodies, nanomedicines, and other macrobiomolecules with varying levels of success. However, clinical application of the pretargeted imaging approach is limited due to the specialized radiochemistry expertise required to synthesize and purify the radiolabeled tag (*e.g.*, radiolabeled tetrazines for bioorthogonal inverse electron demand Diels–Alder reactions). Noncovalent pretargeting approaches such as antibody–hapten and avidin–biotin have been explored in clinical trials but have had a limited success due to immunogenicity.^16^

A new method of pretargeted imaging based on fast and efficient metal complexing affinity of chelators has recently been partially explored for antibodies.^17^ This method involves decoupling of radiometals and bifunctional chelators incorporated on the surface of the antibodies. The tris­(hydroxypyridinone) (THP) bifunctional chelator possesses rapid complexing ability toward Ga^3+^ at chelator concentrations as low as 10 μM and physiological pH.^18,19^ The complex thus formed is highly stable in the presence of serum and in vivo for several hours. The fast chelation and high stability properties of THP have been utilized to perform direct one-step, fast ^68^Ga radiolabeling of small molecules, proteins, and nanoparticles.^12,20–24^ These outstanding radiolabeling properties also allow THP to bind ^68^Ga^3+^ in vivo when administered intravenously to mice previously injected with ^68^Ga despite the dilution of THP conjugates in the blood and the presence of competing proteins such as transferrin and apotransferrin.^17^

Based on these previous results, we hypothesized a possible application of the metal chelation pretargeting strategy toward tracking of liposomal nanomedicines and bone-targeting bisphosphonates. The benefits of this strategy over current approaches include minimal radioactivity handling and requirement for advanced radiochemical expertise, longitudinal tracking over time, and minimal radiation exposure, thereby enabling this approach to be used clinically even in facilities lacking advanced radiochemistry expertise.

To test this pretargeting strategy based on metal chelation, we synthesized a THP-phospholipid conjugate (Figure 1A), which was inserted into preformed PEGylated liposomes to give THP-liposomes (Figure 1B). The presence of the chelator on the liposomal surface acts as a binding site for ^68^Ga, which was first validated in vitro in serum. The serum stability of these radiolabeled liposomes was also determined. These in vitro results were followed by validation of in vivo pretargeting of THP-liposomes (Figure 1C). The same metal pretargeting chelation was also explored with a bone-targeting small-molecule THP-pamidronate^21^ (THP-Pam) to further validate this approach.

**Figure 1:**
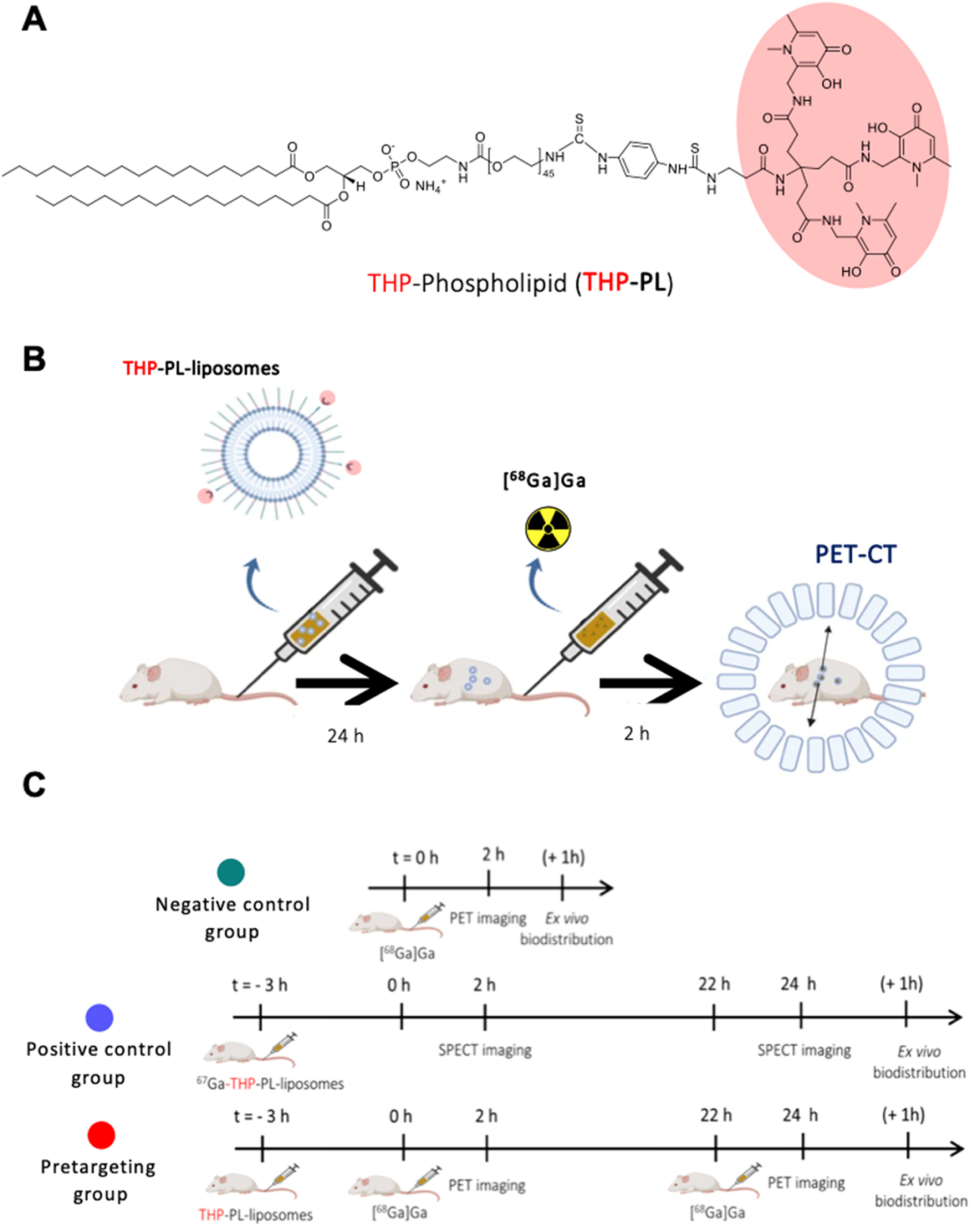
Metal chelation-based pretargeting: (A) structure of THP-phospholipid conjugate used to incorporate THP on the liposomal surface; (B) scheme for metal chelation-based pretargeting of PEGylated liposomes in a murine model using the high chelating affinity of tris­(hydroxypyridinone) toward Ga (Reproduced from Mishra et al., ***RSC Chem. Biol***., 2024, **5**, 622–639); and (C) different imaging groups involved in the metal chelation pretargeting study. Graphics B and C were created using Biorender.

## Materials and Methods

### Materials

All inorganic and organic chemicals were used as received from Sigma-Aldrich, Merck, CheMatech, or Stratech and were of the highest purity grade available. DSPE-PEG(2000) amine (1,2-distearoyl-*sn*-glycero-3-phosphoethanolamine-*N*-[amino­(polyethylene glycol)-2000] ammonium salt) was obtained as a white powder from Avanti Polar Lipids, Inc. (Alabaster, AL) via Merck. THP-Bz-SCN (isothiocyanate derivative of tris­(hydroxypyridinone)) was obtained from CheMatech (Dijon, France) as a white solid. All metal-free chelation reactions were performed in deionized water treated with a Chelex 50 resin. Plain HSPC/Choline/mPEG2000-DSPE-liposomes were obtained from FormuMax Scientific Inc., in a clear glass vial as a translucent whitish liquid. Gallium-68 was eluted in ultrapure HCl (5 mL, 0.1 M, GMP grade from ABX, Germany) as [^68^Ga]­GaCl_3_ from an Eckert & Ziegler ^68^Ge/^68^Ga generator. Gallium-67 was obtained from Guy’s Hospital Radiopharmacy, Guy’s and St Thomas’ NHS Foundation Trust, London, UK as [^67^Ga]­Ga-citrate (5.5 mL, 600 MBq) manufactured for clinical use in patients. Nuclear magnetic resonance (NMR) data was acquired on a Bruker 400 MHz. The obtained spectra were analyzed using MestReNova software. High-resolution mass spectrometry was performed on the National Mass Spectrometry facility at Swansea on Bruker ultrafleXtreme MALDI-TOF/TOF or Autoflex, Bruker Daltonics, at the Mass spectrometry facility at Waterloo campus, King’s College London.

Dialysis was performed using Slide-A-Lyzer G3 Dialysis Cassettes (3.5K MWCO, 3 mL) in 100× volume buffer reservoir. Radio ITLC was developed on an Agilent Technologies silicic-acid-impregnated glass microfiber chromatography paper. Radio instant thin-layer chromatography (ITLC) samples were recorded using a Lablogic Flow-count TLC scanner interfaced with a BioScan B-FC-3200 PMT detector and analysis performed using Laura software. Size-exclusion chromatography (SEC) was performed on a Superose 10/30 column (GE Healthcare Life Sciences) with a flow rate of 0.5 mL/min in PBS buffer. UV detection was performed at 214 and 280 nm on a GE Purifier ÄKTA HPLC. PD MiniTrap G-25 Medium size-exclusion columns containing 2.1 mL of Sephadex resin (GE Healthcare) were used for manual SEC purification. Radioactivity for all samples was measured in a dose calibrator (Capintec. Inc.) or a γ counter (LKB Wallac 1282 Compugamma S) using EdenTerm software. The centrifugation was performed using a Hettich MIKRO 20 benchtop centrifuge. Lyophilization of purified samples was performed using an Edwards Freeze-Dryer Modulyo. Hydrodynamic size and zeta potential were measured on Zetasizer NanoZS from Malvern Panalytical. The mice were scanned in either the preclinical NanoPET/CT imaging system (1:5 coincidence mode; 5 ns coincidence time window) or the NanoSPECT/CT imaging system (Aperture 3:1.2 mm multipinhole, frame time: 83 s, scan time ≈ 1 h) (Mediso Medical Imaging Systems, Budapest, Hungary). The electron microscopy facility at Imperial College London was used to screen the liposome samples under CryoEM.

### Synthesis and Characterization of THP-Phospholipid

For the synthesis of the THP-phospholipid, the reaction was performed between the isothiocyanate derivative of tris­(hydroxypyridinone) and the amine derivative of the DSPE-PEG (2000) phospholipid (Figure S1). THP-Bz-NCS (tris­(hydroxypyridinone) isothiocyanate) (5 mg, 0.0052 mmol) was dissolved in DMSO (250 μL). DSPE-PEG (2000)-amine (1,2-distearoyl-*sn*-glycero-3-phosphoethanolamine-*N*-[amino­(polyethylene glycol)-2000]) (10 mg, 0.0035 mmol) was dissolved in DMSO (250 μL). The phospholipid solution was added to the THP solution along with 5 μL of DIPEA (diisopropyl ethylamine). The reaction was continued at R.T. for 24 h. TLC on silica gel GF (75:36:6 chloroform/methanol/water) was used to monitor the progress of the reaction, showing a new spot below the amino-PEG-DSPE spot due to the formation of the product. The reactant and product were confirmed by charred staining with primuline dye (lipid staining dye). The disappearance of amino-PEG-DSPE (*R*_f_ = 0.76) from the reaction mixture was also confirmed by ninhydrin spray. For purification, the DMSO concentration in the reaction mixture was diluted to 5% using deionized water. The diluted reaction mixture was dialyzed against deionized water (3 × 2000 mL) for over 24 h. The dialysate containing only the product THP-PL (single spot by TLC, MW: 3733 g/mL) was collected and lyophilized. The purified THP-PL was dissolved in deuterated chloroform and a ^1^H NMR spectrumwas obtained. THP-PL was also characterized by high-resolution mass spectrometry (*z* = 2 species in the range 1700–2000 *m*/*z* (peak found at 1866.1; calculated for THP-PL+2H^+^ = 1866.1), the *z* = 3 species in the range 1200–1350 *m*/*z* (peak found at 1245.7; calculated for THP-PL+3H^+^ = 1245.1), and the *z* = 4 species in the range 850–1000 *m*/*z* (peak found at 938.3; calculated for THP-PL+3H^+^+NH_4_^+^ = 938.5)) (Figures S2 and S3).

### Synthesis and Characterization of THP-PL-Liposomes

THP-PL was incorporated into the bilayer of PEG­(2k)-liposomes by equilibrating the Doxebo dispersion (100 μL, 60 mM) and the THP-PL dispersion (0.05 mg, 100 μL) at 50 °C. The equilibrated dispersions were mixed and incubated under constant shaking for 30 min to give THP-PL-liposomes. The formed THP-PL-liposomes were purified using a PD MiniTrap G-25 size-exclusion column (GE Healthcare) following the manufacturer’s gravity protocol. Dynamic light scattering quantified the change in hydrodynamic size, zeta potential, and PDIs of the PEG­(2k)-liposomes pre- and postinsertion reaction. The formed THP-PL-liposomes were also characterized by radiolabeling with ^68^Ga to examine the presence of THP on the surface. For in vivo experiments, THP-PL-liposomes were concentrated by 10-fold using a centrifugal size-exclusion spin filtration (Amicon Ultra 0.5 mL 30 K filters (Millipore, Merck, Germany)).

#### Nanoparticle Tracking Analysis (NTA)

The concentration and hydrodynamic size of the synthesized THP-PL-liposomes were measured by NTA using NanoSight LM10 and NTA software v3.2 (Malvern Panalytical). The stock sample was diluted to achieve ∼100 particles/viewing frame. Measurements were made in triplicates for 60 s with a 488 nm laser for up to three serial dilutions of the sample.

#### Cryoelectron Microscopy

Before sample loading, QUANTIFOIL R 2/2 carbon grids (mesh: Cu 300, #234901; Agar Scientific) were plasma-discharged for 50 s at 30 SCCM gas flow in Nanoclean 1070 (Fischione instruments). 5 μL aliquots of either THP-PL-liposomes or PEG­(2k)-liposomes were deposited on the prepared carbon grids in Vitrobot Mark IV (FEI). The excess liquid was removed by blotting with a filter paper (Agar Scientific) (Parameters: blotting time = 2 s, wait time = 30 s, and blotting force = 2). The grids were instantly frozen in liquid ethane (−188 °C), maintained in liquid N_2_ (−196 °C) in a grid box, and transferred into a cryotransfer holder. CryoEM of these prepared samples was recorded on a TECNAI 12 G2 (FEI) system interfaced with a TemCam-F216 camera and operated using Temmenu v4 software (Tietz Video & Image Processing Systems GmbH, Germany). Parameters for image capture are as follows: electron acceleration = 120 kV, magnification = 52,000×, acquisition time = 1 s, and spot size = 5. Paul Simpson from Imperial College London performed these measurements on the CryoEM.

### Radiochemistry

#### Postprocessing of Generator-Eluted [^68^ Ga]­GaCl_3_

^68^Ga was eluted from a ^68^Ge/^68^Ga E/Z generator with 0.1 N HCl. The peak radioactivity containing 1 mL elution was used for radiolabeling after buffering either with 3.4 M sodium acetate or 1 M sodium carbonate to pH 6.5. Buffered ^68^Ga was used for all radiolabeling and i.v. (intravenous) injections after removal of colloids using saline prerinsed centrifugal filter MW cutoff 50 kDa. RadioTLC was used to characterize the purified ^68^Ga postremoval of colloids (mobile phase (0.175 M citric acid and 0.325 M trisodium citrate in water, unbound ^68^Ga *R*_f_ = 0.7–1; ^68^Ga colloid *R*_f_ = 0)) (Figure S4).

#### Radiolabeling of THP-Phospholipid

The THP-phospholipid conjugate was radiolabeled with ^68^Ga. THP-Bz-SCN, DSPE-PEG (2000)-amine, and ^68^Ga are used as controls. THP-phospholipid (50 μL, 1 mg/mL) was added to ^68^Ga (1–20 MBq, 100–200 μL) and incubated at R.T. for 30 min. RadioTLC on ITLC silica gel GF was performed in mobile phase A (75:36:6 chloroform/methanol/water, unbound ^68^Ga *R*_f_ = 0; [^68^Ga]­Ga-THP *R*_f_ = 0; ^68^Ga colloid *R*_f_ = 0; [^68^Ga]­Ga-THP-PL *R*_f_ = 0.5–0.7) or mobile phase B (0.175 M citric acid and 0.325 M trisodium citrate in water, unbound ^68^Ga *R*_f_ = 0.7–1; [^68^Ga]­Ga-THP *R*_f_ = 0; ^68^Ga colloid *R*_f_ = 0; [^68^Ga]­Ga-THP-PL *R*_f_ = 0–0.1) enabled separation of different radioactive species for qualitative analysis. The radiochemical yield of ^68^Ga radiolabeling was quantified via a PD MiniTrap G-25 size-exclusion column (GE Healthcare) following the manufacturer’s gravity protocol.

#### Radiolabeling of THP-PL-Liposomes

THP-PL-liposomes were radiolabeled with [^68^Ga]Ga at pH 6–7. THP-PL and unmodified PEG­(2k)-liposomes were radiolabeled as controls. The radiolabeling was performed by adding 50 μL (2–10 MBq) of ^68^Ga to either THP-PL-liposomes (200 μL) or unmodified PEG­(2k)-liposomes (200 μL). The radiolabeling reaction mixture was incubated for 30 min. The reaction was purified and the radiochemical yield was quantified via a PD MiniTrap G-25 size-exclusion column (GE Healthcare) following the manufacturer’s gravity protocol.

#### Synthesis of [^67^Ga]­Ga-THP-PL-Liposomes

[^67^Ga]Ga was obtained as citrate, requiring conversion to [^67^Ga]­GaCl_3_ before use in radiolabeling. To this end, [^67^Ga]­Ga-citrate (2 mL, 518 MBq, Guy’s radiopharmacy) was aliquoted in a vial and volume was made up to 5 mL with chelex-treated H_2_O. The whole volume was loaded on a SEP-PAK silica light cartridge multiple times via a syringe until >80% radioactivity was loaded on the column (3 times optimum). Postloading, the loaded column was washed with 5 mL of chelex H_2_O@1 mL min^–1^ thrice. [^67^Ga]­GaCl_3_ is eluted with metal-free HCl (0.1 M, 50 μL). The hottest fractions were buffered to pH 6 and used to radiolabel THP-PL-liposomes, as mentioned above. The radiochemical yield of ^67^Ga radiolabeling was quantified via a PD MiniTrap G-25 size-exclusion column (GE Healthcare) following the manufacturer’s gravity protocol.

### In Vitro Radiochemical Stability

[^68^Ga]­Ga-THP-PL-liposomes (750 μL, 10 MBq, 6 mmol) were incubated at 37 °C in pooled human serum (750 μL) obtained from Sigma-Aldrich. Aliquots (500 μL) of the test sample were taken at different time points for stability study and applied to SEC HPLC at 0, 90, and 180 min. 1 mL fractions were eluted in PBS. UV and radioactivity signal was recorded for each fraction.

The serum stability of [^67^Ga]­Ga-THP-PL-liposomes was also determined using the abovementioned serum stability protocol.

The radiochemical yield of ^68/67^Ga radiolabeling was quantified via a PD MiniTrap G-25 size-exclusion column (GE Healthcare) following the manufacturer’s gravity protocol.

### In Vitro Pretargeting Methods

#### In Vitro ^68^Ga Complexation Test **(i)**

THP-PL-liposomes (200 μL, 4 mM) were added to metal-free human serum (200 μL) obtained from human blood samples postfiltration through BD Gold SST vacutainer tubes and kept under incubation at 37 °C for 60 min. Buffered ^68^Ga (5 MBq, 50 μL) was added to serum-incubated THP-PL-liposomes and incubated at 37 °C. Aliquots of the incubated reaction were collected at 30 min, applied to SEC HPLC, eluted with PBS in 30 fractions of 1 mL, and measured for UV signal and radioactivity.

#### In Vitro ^68^Ga Complexation Test **(ii)**

Buffered ^68^Ga (5 MBq, 50 μL) was added to metal-free human serum (200 μL) obtained from human blood samples postfiltration through BD Gold SST vacutainer tubes and kept under incubation at 37 °C for 60 min. THP-PL-liposomes (200 μL, 4 mM) were added to serum-incubated ^68^Ga and incubated at 37 °C. Aliquots of the incubated reaction were collected at 30 min, applied to SEC HPLC, eluted with PBS in 30 fractions of 1 mL, and measured for UV signal and radioactivity.

### In Vivo PET Imaging

#### Animals

All animal experiments were ethically approved by the Animal Welfare & Ethical Review Board at King’s College London and the experiments were carried out in accordance with the Animals (Scientific Procedures) Act 1986 (ASPA) UK Home Office regulations governing animal experimentation. The healthy animal studies were performed under project license PPL PBBA9A243. All in vivo experiments were conducted on healthy female BALB/c mice (8–9 weeks old) sourced from Charles River UK Ltd.

#### Preclinical PET/CT and SPECT/CT Scanners

Each mouse (*n* = 3–5, BALB/c, female, aged 6–8 weeks, 17–20 g body weight) was anesthetized by inhalation of isoflurane (2–3% in oxygen). The tracer/molecule of interest was injected via tail vein intravenous injection. The mouse was scanned in either a nanoscan in vivo preclinical PET/CT imaging system (1:5 coincidence mode; 5 ns coincidence time window) or a SPECT/CT imaging system (start frame: 3 mm; end frame: 107 mm; frame time: 83 s, scan time: ∼1 h) (Mediso Medical Imaging Systems, Budapest, Hungary) depending on the tracer injected. PET/CT images were reconstructed using Tera-Tomo 3D reconstruction (400–600 keV energy window, 1–3 coincidence mode, 4 iterations and subsets, voxel size 0.4 × 0.4 × 0.4 mm^3^) and corrected for attenuation, scatter, and decay. The data were binned into 17 frames (1 × 1, 10 × 3, 5 × 5, and 1 × 4 min) for dynamic analysis. For SPECT images, reconstruction was performed using the HiSPECT standard method.

### Pretargeting of THP-PL-Liposomes in Healthy Animals

For negative control group 1, the mice (*n* = 3) were anesthetized and injected intravenously through the tail vein with purified ^68^Ga (10 MBq, 100 μL). The mice were kept anesthetized for 2 h postinjection and then imaged via an in vivo preclinical PET/CT imaging system.

For pretargeting test group 2, the mice (*n* = 4) were anesthetized and injected intravenously through the tail vein with THP-PL-liposomes (50 mM lipid concentration, 100 μL) at *t* = −3 h. At *t* = 5 h, mice were anesthetized and injected intravenously through the tail vein with purified ^68^Ga (10 MBq, 100 μL). The mice were kept anesthetized for 2 h postinjection and then imaged via an in vivo preclinical PET/CT imaging system. At *t* = 22 h, mice were anesthetized and injected intravenously through the tail vein with purified ^68^Ga (10 MBq, 100 μL). The mice were kept anesthetized for 2 h postinjection and then imaged via an in vivo preclinical PET/CT imaging system.

For positive control group 3, the mice (*n* = 6) were anesthetized and injected intravenously through the tail vein with ^67^Ga-THP-PL-liposomes (3 MBq, 50 mM lipid concentration, 100 μL) and imaged at *t* = 2 h, 24 h via a preclinical SPECT-CT imaging system. For the blood kinetic study, blood was withdrawn from a puncture on the tail vein using 20 μL capillary tubes at 3, 6, 9, 22, and 25 h post administration of liposomes. The collected blood was measured and counted for radioactivity and %IA/g was determined at each time point.

The mice were euthanized postscan by cervical dislocation and organs of interest were collected, weighed, and measured for radioactivity along with the serial dilution of injected radiotracer standards for the determination of ex vivo biodistribution in percentage injected activity per gram of tissue (% IA/g).

### Pretargeting of Bone-Targeting THP-Pam in Healthy Animals

The synthesis and radiolabeling of THP-Pam are performed as described here.^21^ The negative control group (free ^68^Ga) from the liposomal pretargeting study also acted as the negative control for the pretargeting of the THP-Pam study.

For the THP-Pam in vivo labeling group, the mice (*n* = 4) were anesthetized and injected i.v. with THP-Palmidronate (50 ug in 100 μL saline) at *t* = −3 h. At *t* = 0 h, mice were anesthetized and injected i.v. with purified ^68^Ga (10 MBq, 100 μL). The mice were kept anesthetized for 2 h postinjection and then imaged via an in vivo preclinical PET/CT imaging system. At *t* = 24 h, mice were anesthetized and injected i.v. with ^68^Ga (10 MBq, 100 μL). The mice were kept anesthetized for 2 h postinjection and then imaged via an in vivo preclinical PET/CT imaging system. The mice were euthanized postscan by cervical dislocation and the required organs were collected, weighed, and measured for radioactivity in a γ counter.

For the THP-Pam positive control group, the mice (*n* = 4) were anesthetized and injected with radiolabeled ^68^Ga-THP-Pam (22 MBq, 100 μL), which was imaged at 1 h p.i.

The mice were euthanized postscan by cervical dislocation and the required organs were collected, weighed, and measured for radioactivity in a γ counter. Serial standard dilution of the injected radiotracer was measured alongside the collected organs to calculate the percentage injected activity per gram of tissue (%IA/g).

### Analysis

All numerical data were plotted and analyzed using either GraphPad Prism 8 or advanced versions. Data are presented as mean ± standard deviation (SD) unless stated otherwise. All statistical tests performed are either multiple paired or unpaired *t* tests to determine significant differences among test and control groups.

The analysis of the reconstructed in vivo images was performed using VivoQuant 3.5 (Perceptive formerly known as Invicro Inc.). Regions of interest (ROI) were drawn over the knees for bones and the heart as a measure of the blood pool, kidneys, lungs, bladder, spleen, liver, muscles, and brain in PET and SPECT images for image quantification. The data from these ROIs was collected in %IA/g, where %IA was determined by the amount of radioactivity administered.

Additionally, for SPECT quantification, dimensionless image count values were converted to units of radioactivity concentration in megabecquerel (MBq) units by the InVivoScope analysis software incorporating a user-defined calibration factor stored within the software. This calibration factor was experimentally predetermined for the isotope used (gallium-67) and the 1.2 mm aperture (same aperture used for animal imaging) using a syringe filled with a known concentration of activity previously measured at a known time in a dose calibrator. The syringe was then imaged, acquiring a minimum of 1,00,000 counts per frame, and the scan was reconstructed to determine the calibration factor.

## Results and Discussion

### Synthesis and Characterization of THP-PEG2000-DSPE (THP-PL)

To provide gallium-binding properties to PEGylated liposomes, a chelator-PEG-phospholipid conjugate based on tris­(hydroxypyridinone) (THP) was synthesized (THP-PL). THP was selected over other gallium chelators for its proven high affinity and fast complexation kinetics, even in the presence of competing metal ions,^18^ while NH_2_-PEG2000-DSPE was chosen as PEG2000-DSPE is the basic phospholipid component of stealth liposomes. Different modified versions of PEG2000-DSPE have been used routinely to form active targeting liposomes with surface modifications via embedding the modified phospholipid conjugate in the bilayer of the liposomes.^25^ The PEG chain length of 2000 (∼45 repeating units, 2000 g/mol) was chosen to avoid burying the THP chelators among PEG chains on the surface of the liposomes and keep them accessible for interaction with the radiometal.

The desired THP-PL conjugate was obtained from the reaction of NH_2_-PEG2000-DSPE with 1.5 equiv excess of the isothiocyanate conjugate of THP (THP-Bz-SCN) in the presence of diisopropyl ethylamine (DIPEA) as a base, forming a thiourea bond. The thiourea bond thus formed has been efficiently used to obtain THP-based radiotracers, which have shown to exhibit stability in vivo.^12,21,26,27^ The dialysis purification of the reaction mixture provided THP-PL as a white light powder with a yield of 84 ± 6%. NMR and HR-MS confirmed the structure of the obtained conjugate (Figures S2 and S3). Moreover, the formation of the thiourea bond was evidenced by the disappearance of the −NH_2_ peak at δ 1.69 ppm in the ^1^H NMR spectrum and the appearance of thiourea protons at ca. δ 9 ppm as broad peaks. Integration of peaks could not be performed due to the high-intensity ethylene peak of PEG at δ 3.64 ppm (Figure S2). High-resolution electrospray ionization mass spectrometry (ESI-MS) further confirmed the formation of THP-PL. The spectrum of THP-PL (THP-PL exact MW = 3733) showed the expected electrospray envelope nature of PEG polymers and the *z* = 2 species in the range 1700–2000 *m*/*z* (peak found at 1866.1; calculated for THP-PL = 1866.1), the *z* = 3 species in the range 1200–1350 *m*/*z* (peak found at 1245.7; calculated for THP-PL = 1245.1) (Figure S3). These peaks were not observed in the ESI-MS of PL performed under identical conditions.

### ^68^Ga Radiolabeling of THP-PL

Having obtained spectroscopic confirmation of the success of the conjugation reaction, the binding affinity of THP-PL toward gallium was tested by radiolabeling this new phospholipid with ^68^Ga. The radiolabeling reaction was performed by eluting ^68^Ga from the generator with 0.1 M HCl, followed by neutralization of an aliquot with concentrated sodium carbonate to pH 6, and mixing with an aqueous solution of THP-PL at room temperature for 30 min (final buffer concentration = 30 mM carbonate, final THP-PL concentration = 50 μM). These reaction conditions were chosen as they have been shown to exceed the minimum concentration and reaction times required for efficient ^68^Ga reaction with THP (submicromolar ligand concentrations and 5 min are sufficient for >95% RLY with other THP-containing molecules).^18^ The radiolabeling yield of THP-PL was compared to that of PL, THP, and buffered ^68^Ga as controls (Figure 2A,B). The analysis of ^68^Ga radiolabeling of synthesized THP-PL and controls by radioTLC showed high radiolabeling yields. Two solvent systems were used as mobile phases to confirm this result. Using citrate buffer as a mobile system facilitated separation between PL and THP-PL species, whereas using a 75:36:6 chloroform/methanol/water mixture facilitated separation between THP and THP-PL (Figure 2A,B). However, the high molecular weight of THP-PL restricted the complete migration of the conjugate on the TLC plate, thereby preventing the quantification of radiochemical yield. Quantification of radiolabeling was achieved using size-exclusion chromatography, allowing complete separation of THP-PL and unreacted ^68^Ga, giving a nonoptimized radiolabeling yield of 76 ± 4%.

**Figure 2:**
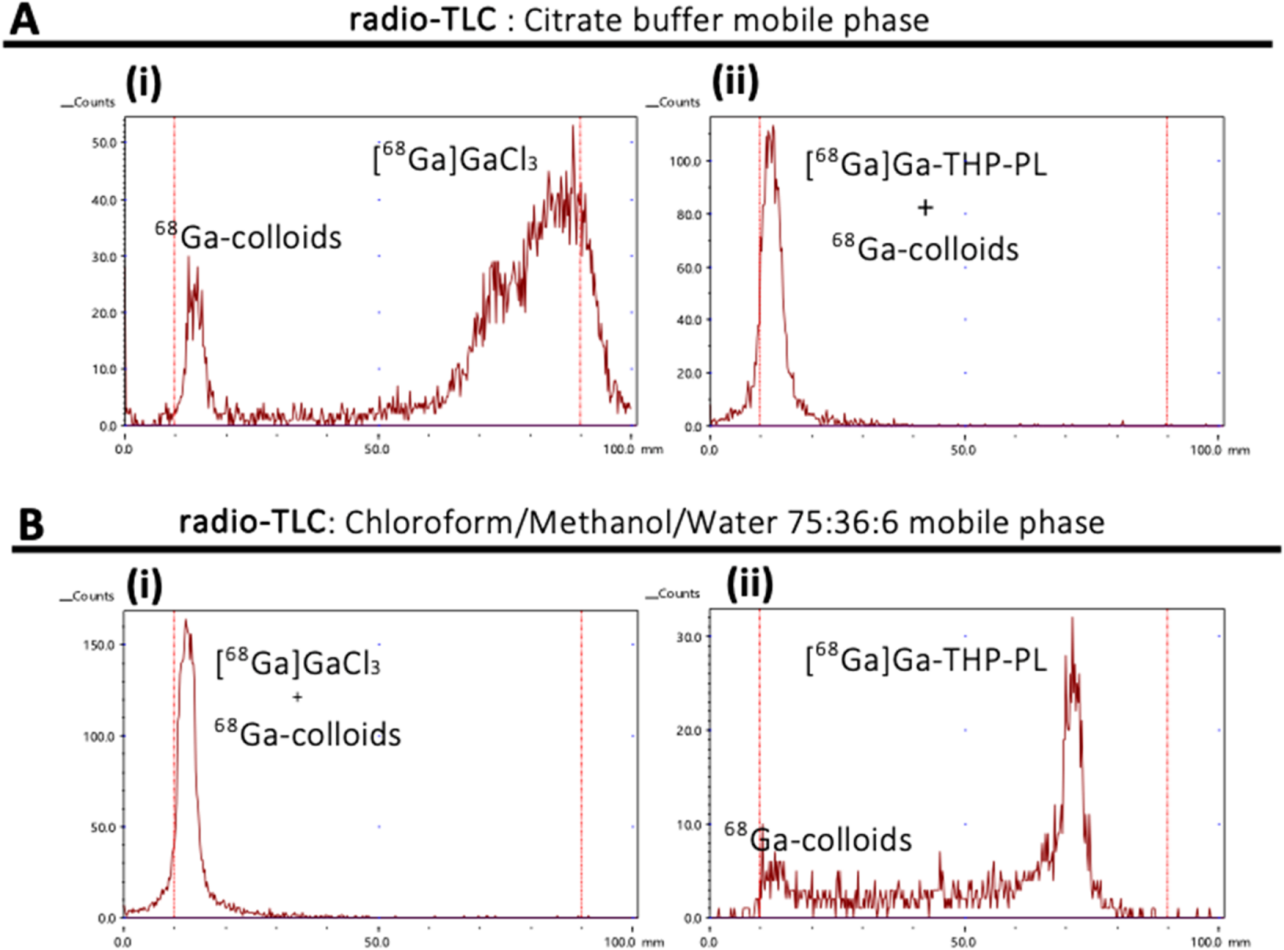
Radiochemistry of THP-PL. (A, B) Radioanalysis of [^68^Ga]­Ga-THP-Pam and unbound ^68^Ga as a comparison: (A) ITLC of unbound ^68^Ga (i) and [^68^Ga]­Ga-THP-PL (ii) in 0.5 M citrate buffer pH 5.5; (B) ITLC of unbound ^68^Ga (i) and [^68^Ga]­Ga-THP-PL (ii) in chloroform:methanol:water (ratio 75:36:6).

### Synthesis and Characterization of THP-PL-Liposomes

THP-PL was inserted into the lipid bilayer of PEGylated liposomes using a standard protocol (Figure 3A), previously shown to allow the insertion of phospholipids without significantly modifying the original properties of PEGylated liposomes.^12,28^ The PEGylated liposomes used were chosen for having similar physicochemical properties as stealth liposomes extensively used in the clinic, such as Doxil/Caelyx (size: 89.4 ± 0.6 nm, zeta potential: −0.7 ± 0.6 mV, lipid concentration: 60.0 ± 0.9 mM). After the insertion reaction—that involved coincubation of THP-PL and PEG­(2k)-liposomes at 50 °C for 30 min—the THP chelator was expected to be accessible on the liposome surface, hence retaining its chelation affinity toward gallium. To facilitate this, a key design concept of THP-PL was to contain a PEG chain of the same molecular weight as the PEG chains present in PEG­(2k)-liposomes (i.e., 2000 Da). The insertion procedure was performed with 0.5% mole percent of inserted phospholipid to total phospholipid. This mole percent, according to previous studies,^29^ is known to provide 90% insertion success, allowing for the average incorporation of up to ca. 500 THP chelators on the surface of each liposome. Attempts were made to confirm the experimental chelator concentration on the surface of the liposomes using UV–vis titrations with iron­(III) and gallium­(III); however, these attempts were unsuccessful.

**Figure 3:**
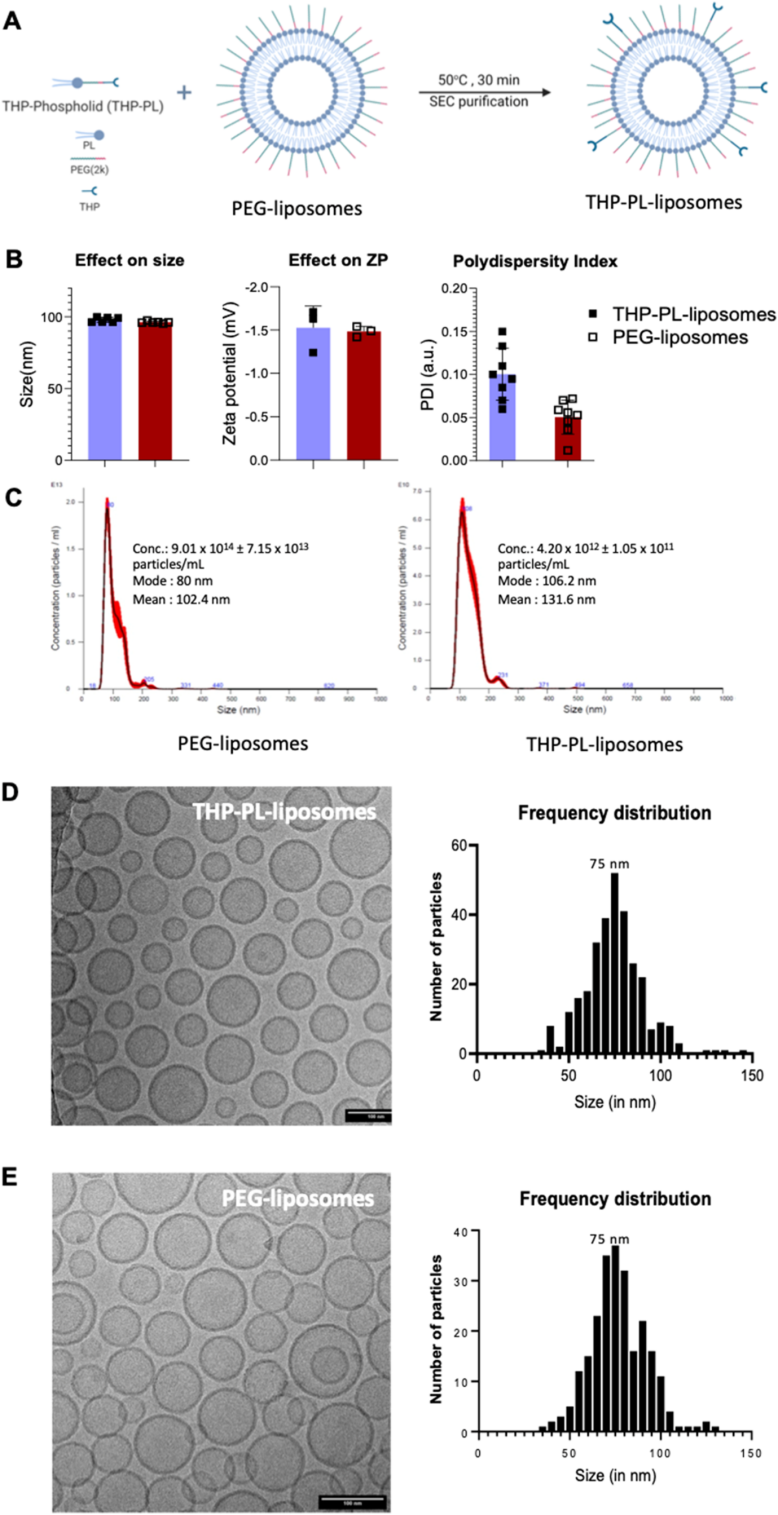
Synthesis and characterization of THP-PL-liposomes: (A) insertion of THP-PL in PEGylated liposomes by coincubation of THP-PL and PEG-2k liposomes; (B) hydrodynamic size of THP-PL-liposomes and PEG­(2k)-liposomes confirmed no effect on size (Z-average) due to insertion of THP-PL into the liposomes; zeta potential (ZP) measurement of the liposome samples showed minimal change in the magnitude of surface charge with no change in the polarity postinsertion of THP-PL in the liposomal bilayer; the polydispersity index (PDI) was unaffected postinsertion (data presented as mean ± SD, *n* = 3–8); (C) nanoparticle tracking analysis (NTA) results: PEG­(2k)-liposomes (concentration: 9.01 × 10^14^ ± 7.15 × 10^13^ particles/mL, mode:80 nm, mean: 102.4 nm); THP-PL-liposomes (concentration: 4.20 × 10^12^ ± 1.05 × 10^11^ particles/mL, mode: 106.2 nm, mean: 131.6 nm); (D, E) Cryoelectron microscopy of liposomes pre- and surface postmodification with THP-PL: (D) THP-PL-liposomes have retained their spherical nature and the size distribution is not altered by insertion of THP-PL into the bilayer of liposomes and the bilayer nature is also retained; (E) PEG­(2k)-liposomes spherical morphology and bilayer structure. Graphic A was created using Biorender.

To assess the success of the insertion reaction, the product (THP-PL-liposomes) was purified by size exclusion (PD10 miniTrap G-25 size-exclusion column) and analyzed via radiolabeling and dynamic light scattering (DLS) studies. Both the hydrodynamic size and zeta potential (a measure of surface charge for nanoparticles) were unaffected by the modification of liposomes (Figure 3B). The hydrodynamic size (z-average) was not affected by the insertion of the THP-PL: 97 ± 3 nm (unmodified liposomes) and 95 ± 2 nm (THP-PL-liposomes). The surface charge of the liposomes (*z*-potential) was measured since it plays a key role in their in vivo behavior. These measurements showed no effect of the THP-PL insertion in the zeta potential: −5.2 ± 1.3 mV (PEG­(2k)-liposomes) and −5.7 ± 0.9 mV (THP-PL-liposomes). Finally, the polydispersity index (PDI) was also measured. PDI is a measurement of the number of species of a particular size providing information on the monodispersity of the sample. Clinical-grade liposomes are highly monodispersed to minimize the variability in the injected samples. FDA’s guidelines suggest that PDI should remain below 0.3.^30^ The PDI of the THP-PL-liposomes was 0.10 ± 0.03 and that for the PEG­(2k)-liposomes was 0.05 ± 0.02. Taken all together, these results confirmed that the insertion reaction had no major impact on the surface charge (zeta potential), polydispersity, and hydrodynamic size.

The determination of liposome concentration was performed using nanoparticle tracking analysis (NTA) as concentration plays an important role in the in vivo clearance of liposomes. The liposome concentration acts as an indirect measurement of lipid concentration, which needs to be maintained above 4 μmol/mouse to avoid fast clearance and maintain their characteristic long blood circulation. The liposome concentration for the PEG­(2k)-liposome was 9.01 × 10^14^ ± 7.15 × 10^13^ particles/mL and that for the THP-PL-liposome was 4.20 × 10^12^ ± 1.05 × 10^11^ particles/mL (Figure 3C). This observation confirmed expected loss of liposomes during modification and purification steps and therefore, for in vivo experiments, the THP-PL-liposomes were concentrated and doped with 10% PEG­(2k)-liposomes to maintain the lipid concentration above 4 μmol/mouse. The size distribution of both liposome samples using NTA was 80–200 nm with peak maxima at 80 and 106 nm for PEG­(2k)-liposomes and THP-PL-liposomes, respectively. In both samples, the liposomal size is concentrated toward the ∼100 nm region with a small shoulder representing the particles with a slightly larger size greater than 100 nm.

THP-PL-liposomes and PEG­(2k)-liposomes were also analyzed by cryotransmission electron microscopy (cryo-TEM) to examine if the insertion of THP-PL had caused any modifications to their shape, bilayer structure, and size distribution. The THP-PL-liposomes remained unaltered as observed in Figure 3D,E. 50 representative microscopic images were taken for each sample to confirm the above observations. Analysis of these microscopic images using ImageJ allowed us to create a size distribution shown as a histogram in Figure 3D,E. Thereby, cryo-TEM along with the data from DLS and NTA confirmed that nonsignificant change in the liposomal size, PDI, and zeta size and no change in the shape and structure of the liposomes was observed postmodification.

### ^68^Ga Radiolabeling of THP-PL-Liposomes

To confirm the presence of the chelator and its accessibility/reactivity toward ^68^Ga, the THP-PL-liposomes were reacted with buffered ^68^Ga for 30 min and passed through a size-exclusion column to collect the labeled THP-PL-liposomes and unreacted ^68^Ga. PEG­(2k)-liposomes and THP-phospholipid were radiolabeled and subjected to the same size-exclusion chromatography purification method as controls. The THP-PL-liposomes were labeled with an RLY of 94.6 ± 1.9% (Figure 4A), whereas PEG­(2k)-liposomes only showed a RLY of 3.4 ± 0.8%, demonstrating the success of the THP-PL insertion reaction. In summary, this data indicated that THP-PL has been successfully incorporated into PEG­(2k)-liposomes, with no major impact on the original liposomal properties.

**Figure 4:**
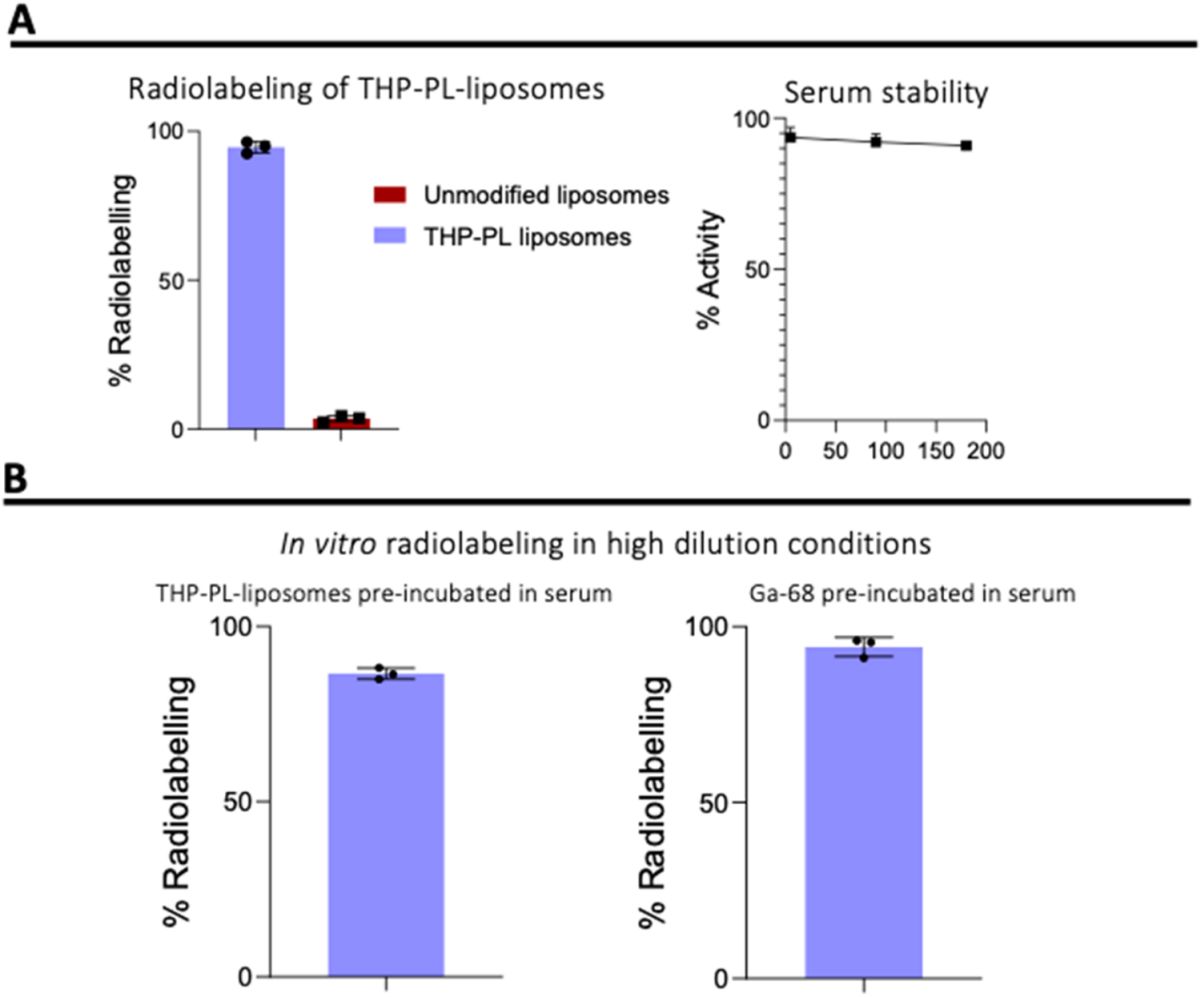
Radiochemistry of THP-PL-liposomes. (A) THP-PL-liposomes showed % radiolabeling of 94.6 ± 1.9% compared to <5% nonspecific radiolabeling of unmodified PEGylated liposomes with a serum stability of 90.9 ± 0.6% in human serum after 180 min; (B) (left) in vitro pretargeting of THP-PL-liposomes with ^68^Ga incubated in citrate-free human serum; (right) in vitro pretargeting of THP-PL-liposomes incubated in citrate-free human serum with ^68^Ga. Data presented as mean ± SD, *n* = 3 in all cases.

### *In Vitro* Chelation of ^68^Ga by THP-PL-Liposomes in the Human Serum

One of the main challenges of our proposed in vivo radiolabeling strategy is that radiometal binding to the liposome must occur in the presence of many potential competitors. It is well-established that free Ga^3+^ binds to the Fe^3+^-transport protein serum transferrin (Tf), present in high concentrations in blood.^31,32^ To assess the ability of ^68^Ga to preferentially bind THP-PL-liposomes in the presence of serum components, two in vitro radiolabeling experiments were performed: (i) preincubation of THP-PL-liposomes with human serum for 1 h at 37 °C, followed by the addition of ^68^Ga; and (ii) preincubation of ^68^Ga with human serum for 1 h at 37 °C, followed by the addition of THP-PL-liposomes. In both cases, size-exclusion chromatography was used to isolate and quantify liposome-bound ^68^Ga radioactivity from that bound to serum components. In (i), THP-PL-liposomes were successfully radiolabeled with a high efficiency of 94 ± 2% (*n* = 3) in 15 min post addition of ^68^Ga (Figure 4B). In (ii), THP-PL-liposomes were radiolabeled with a high efficiency of 87 ± 2% (*n* = 3) (Figure 4B). These results further confirmed the high affinity of the THP chelator toward ^68^Ga within a biologically relevant environment.

### *In Vitro* Stability of ^68^Ga/^67^Ga-THP-PL-Liposomes

It has been previously demonstrated that ^68^Ga/^67^Ga-THP complexes are highly stable in vivo, both in animals and humans, with no signs of demetalation.^18–24,26,33^ This was an essential factor in the design of our strategy, as any release of free gallium from the liposomes could undermine the proposed in vivo radiolabeling approach. To provide further support before in vivo imaging studies, we used size-exclusion chromatography to test the radiochemical stability of ^68^Ga/^67^Ga-THP-PL-liposomes in vitro in human serum at 37 °C. Using this system, both liposomes and serum components can be efficiently separated and quantified via UV and radioactivity measurements, allowing the determination of liposome and serum protein-associated radioactivity at different time intervals. ^68^Ga-THP-PL-liposomes showed high stability in human serum with 91 ± 1% of the radioactivity associated with the liposomes over 3 h (Figure 4A). This high stability was retained for longer periods, as demonstrated by the >94% radiochemical stability found for ^67^Ga-THP-PL-liposomes after 48 h under the same conditions.

### In Vivo PET/SPECT Imaging

Following successful synthesis and characterization of the components of the system, we performed in vivo PET imaging and biodistribution experiments. A critical variable in the development of an in vivo labeling system is the duration between the introduction of the liposomes and the administration of the radioisotope. This time point was determined from our previous liposomal imaging studies^34,35^ —including in vivo pretargeting using standard bioorthogonal chemistry^12^—and imaging of ex vivo prelabeled ^67^Ga-THP-PL-liposomes (henceforth addressed as the *positive control group*).

The positive control group (prelabeled ^67^Ga-THP-PL-liposomes; Figure 5) shows the biodistribution of THP-PL-liposomes. The radioactivity was observed in the liver, spleen, and bloodstream including circulatory vasculature such as the heart, carotid arteries, and aorta at *t* = 2 h. At *t* = 24 h p.i. (post intravenous administration), the activity had cleared from the blood and concentrated mainly in the liver and spleen (Figure 5B). The one-phase decay fit of the blood kinetics of liposomes, as observed in Figure 5D, showed initial blood clearance half-life ^67^Ga-THP-PL-liposomes as *t*_1/2_ = 7.9 h with >11%IA/g still in circulation 25 h p.i. This long circulation and slow and steady accumulation in the spleen and liver is typical of PEGylated liposomal nanomedicines such as Doxebo, as PEG is known to inhibit recognition by the reticuloendothelial system (RES).^2^ However, the blood clearance of these liposomes was comparatively faster than observed for Doxil. This could be either attributed to minor differences in the structure of these empty PEGylated liposomes used in our study that do not carry a drug in the intraliposomal space or the presence of THP-PL on the surface of the bilayer on account of insertion.^2,36^ The liposome concentration in blood was reduced to less than a quarter of their value at *t* = 25 h (8.2 ± 3.7%IA/g) from the initial time point of administration at *t* = 0 h (38.9 ± 7.3%IA/g). Thus, 24 h post liposomal injection, when the THP-PL-liposomes are partially in blood circulation and accumulated in solid organs (i.e., liver and spleen), was chosen as a time point for investigation in our pretargeting experiments (*vide infra*). In addition, 5 h post liposome injection for imaging was chosen to test in vivo radiolabeling when the largest fraction of the liposomes are circulating in the bloodstream.

**Figure 5:**
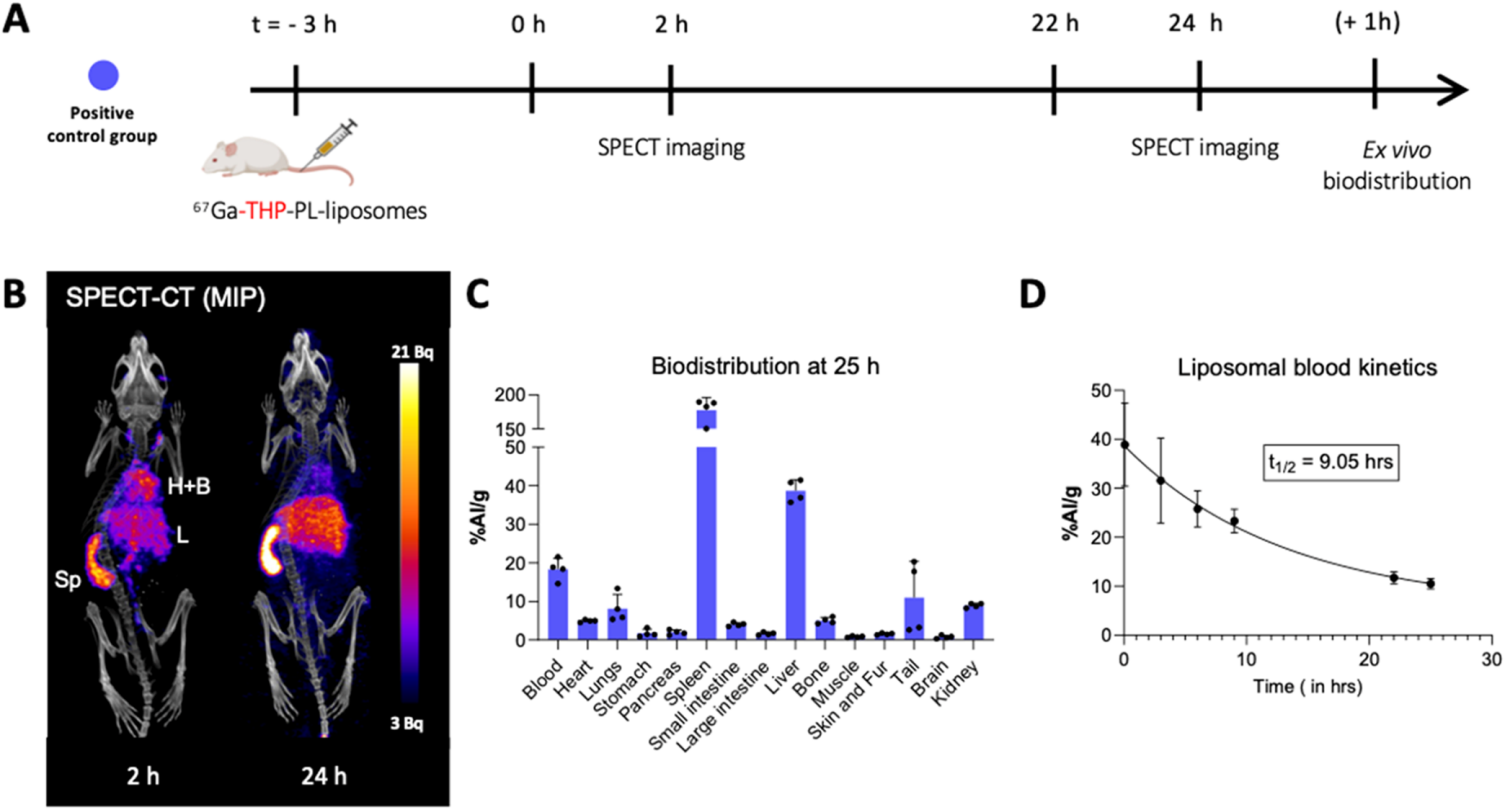
In vivo imaging: Positive control group: (A) schematic of the in vivo imaging positive control group; (B) representative in vivo SPECT image for the positive control group *i.e*.,^67^Ga-THP-PL-liposome (3 MBq, 50 mM lipid concentration, 100 μL) at *t* = 2 and 24 h (H—heart, B—blood, L—liver, Sp—spleen); (C) biodistribution at *t* = 25 h (*n* = 4); (D) blood kinetics in vivo study to determine the circulation characteristics of ^67^Ga-THP-PL-liposomes injected intravenously (*n* = 4). Data is presented as mean ± SD, *n* = 4 in all cases. Graphic A was created using Biorender.

The *negative control group* consisted of free ^68^Ga administered and imaged at *t* = 2 h with ex vivo biodistribution performed postimaging (Figure 6). Free ^68^Ga was quickly cleared from the blood pool via the kidneys leading to high radioactivity in the urinary bladder. Uptake was also observed in bones (especially joints) and gut post *t* = 2 h. The acetate-neutralized ^68^Ga showed very similar in vivo behavior to ^68^Ga-citrate, which can be explained on account of acetate, like citrate, being a weak chelator for ^68^Ga.^37–39^ The administered ^68^Ga ion is likely to transchelate from its acetate salt form and bind to transferrin, lactoferrin, and other iron-binding proteins,^40,41^ and explaining high blood pool values of ∼10% ID/g even after 3 h and also being responsible for accumulation observed in the lungs, bones, and the small intestine.^42^

**Figure 6:**
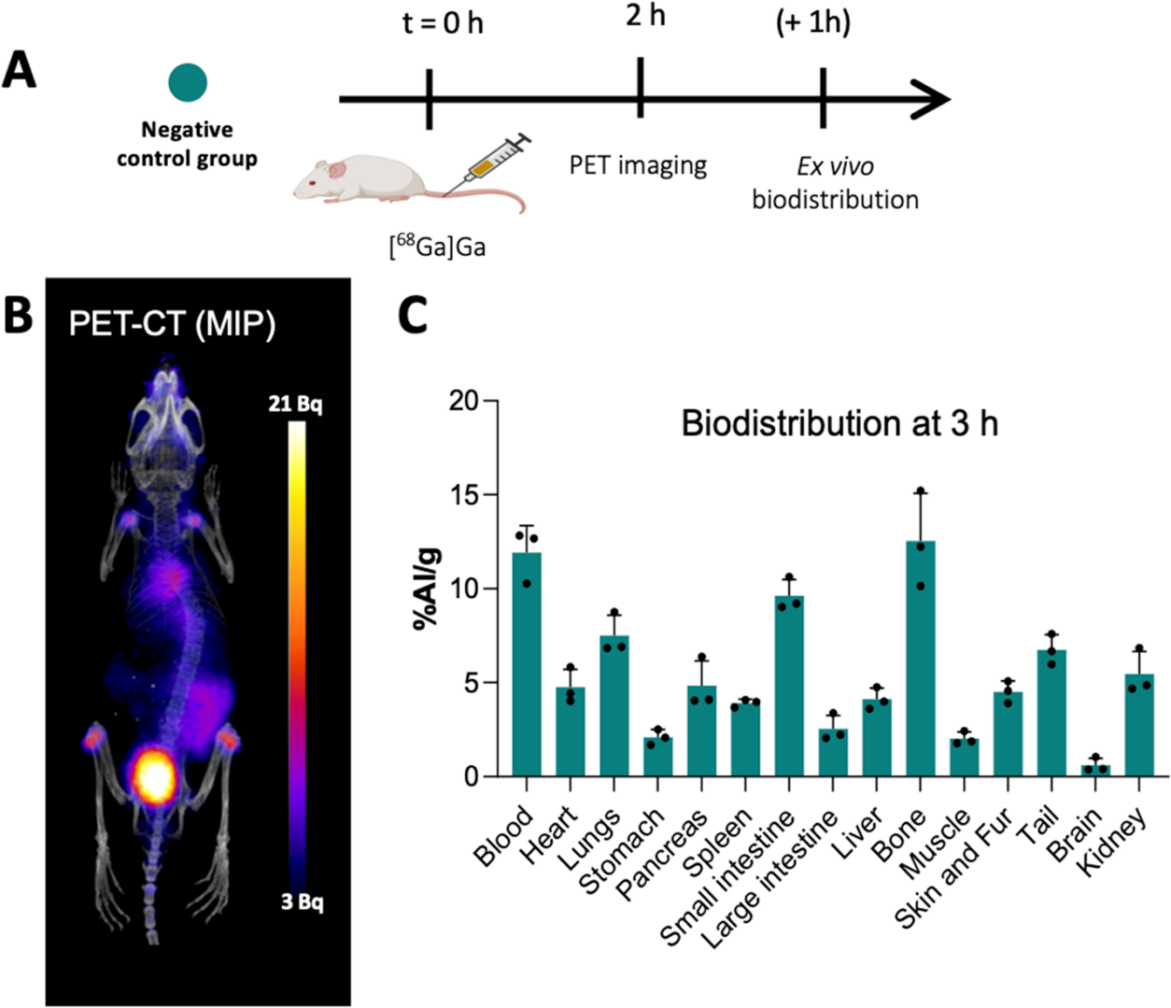
In vivo imaging: negative control group: (A) schematic of the in vivo imaging negative control group; (B) representative in vivo PET image for the negative control group *t* = 2 h postinjection of buffered unbound ^68^Ga (10 MBq, 100 μL) (H—heart, B—blood, Int—intestine, Bla—bladder); (C) biodistribution at *t* = 3 h (*n* = 3). Data is presented as mean ± SD, *n* = 3 in all cases. Graphic A was created using Biorender.

The *pretargeting group* involved PET imaging at two time points: *t* = 2 and 24 h (Figure 7). At *t* = 2 h, the radioactivity signal was observed in the blood pool (e.g., heart, carotid arteries, and aorta), and low uptake was observed in the liver and spleen. This indicates in vivo labeling of the THP-PL-liposomes that are circulating in the blood pool, as observed in the *positive control group* (Figure 5B–D). At *t* = 24 h, the activity was observed in the bones and urinary bladder with limited uptake in blood showing limited in vivo labeling of blood circulating liposomes, which was also confirmed by the biodistribution data. At this late time point, the PET image, biodistribution, and image quantification showed similarities with the negative control group (Figure 6). However, ex vivo quantification showed higher uptake of ^68^Ga in the blood (14.6 ± 0.4 vs. 11.9 ± 1.4%IA/g), heart (7.6 ± 1.5 vs 4.7 ± 0.9%IA/g), and spleen (6.4 ± 0.6 vs. 3.9 ± 0.2%IA/g) for the pretargeting group compared to the negative control, which can be attributed to in vivo labeling of liposomes even at 24 h after THP-PL-liposome administration.

**Figure 7:**
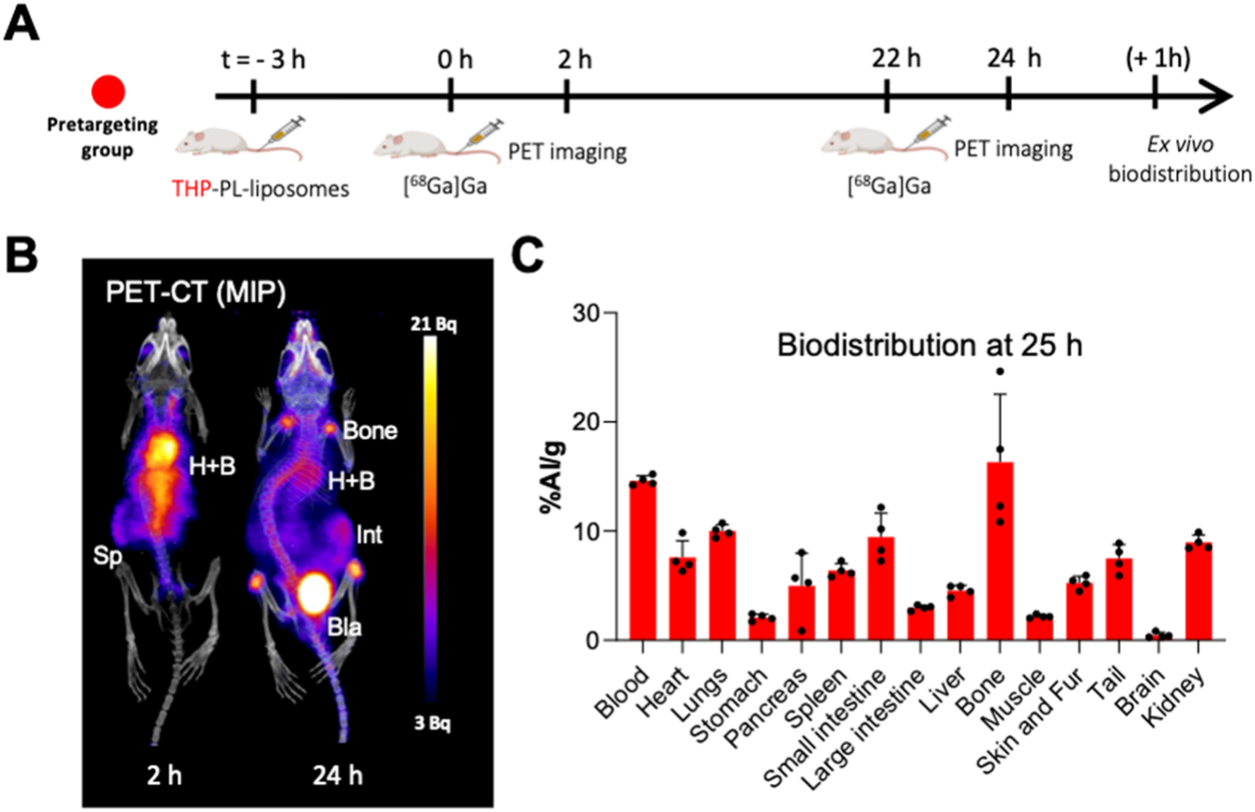
In vivo imaging: pretargeting group: (A) schematic of the in vivo pretargeting group experiment; (B) representative in vivo PET pretargeted images of THP-PL-liposome at *t* = 2 and 24 h. THP-PL-liposomes (50 mM lipid concentration, 100 μL), ^68^Ga (10 MBq, 100 μL) (H—heart, B—blood, Int—intestine, Bla—bladder, Sp—spleen); (C) biodistribution at *t* = 25 h (*n* = 4). Data is presented as mean ± SD, *n* = 4 in all cases. Graphic A was created using Biorender.

These in vivo PET results showed that labeling of THP-PL-liposomes with unchelated ^68^Ga occurs in the blood pool. At the time point of *t* = 2 h, the liposomes were mainly in circulation and were labeled by ^68^Ga. At the later time point (i.e., *t* = 24 h), however, the images and biodistribution suggest minimal gallium chelation by THP-PL-liposomes and clearance from the blood via the urinary system, thereby showing a high bladder uptake of 45.7 ± 10.8%IA/g. Further comparison of radioactivity uptake for each organ within different groups at different time points was performed as seen in Figure 8 and also plotted as a heat map and can be found in Figure S5. This heat map again shows how pretargeting works effectively at earlier time points when most of the THP-PL-liposomes are circulating in the blood. The heat map for a later time point confirmed minimal pretargeting with the administered ^68^Ga in the pretargeting group showing a fate similar to the negative control.

**Figure 8:**
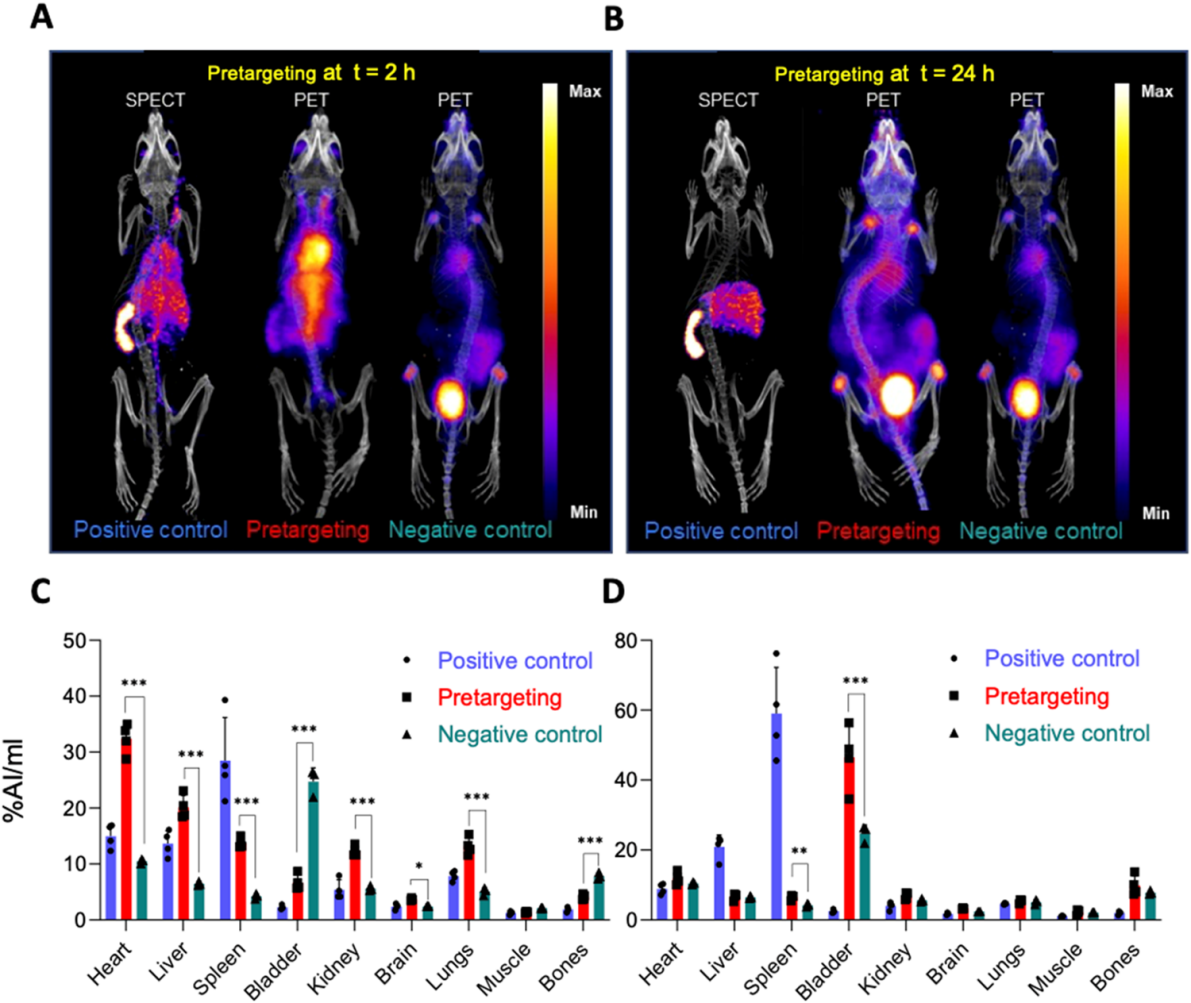
Comparison between different in vivo groups at two pretargeting time points for THP-PL-liposomes: (A, B) THP-PL-liposome pretargeting PET/SPECT scans at two different time points of interest (A) at *t* = 2 h and (B) at *t* = 24 h; (C, D) comparison of image quantification performed on different in vivo groups for THP-PL-liposome pretargeting PET/SPECT images by drawing ROIs using VivoQuant (*n* = 4) at two different time points of interest (C) at *t* = 2 h and (D) at *t* = 24 h. Data are presented as mean ± SD, *n* = 3–4 in all cases. Unpaired *t* test comparisons were performed pairwise on each organ per group to determine significance (*, *P* < 0.05; **, *P* < 0.005; ***, *P* < 0.001).

The observation of low in vivo labeling at the later time point may be due to several reasons: First, the saturation of the THP chelator on the liposomal surface by transchelation of iron from blood proteins such as hemoglobin, transferrin, and ferritin; second, the hydrophilicity of ^68^Ga (injected as one of the components of the in vivo labeling system) can hinder its penetration in tissues and reach organs such as the liver and spleen, which are known to be passive targets for liposomes in healthy subjects as seen in the positive control group ^67^Ga-THP-PL-liposomes. Finally, the other possible explanation behind the low pretargeting could be the inaccessibility of the THP-PL-liposomes for pretargeting due to uptake by cells.

In summary, the in vivo labeling between preinjected THP-PL-liposomes and free ^68^Ga was observed successfully at earlier time points when most of the liposomes are circulating in the blood, leading to imaging of the blood pool. However, at the later time point of interest, i.e., 24–48 h, when the liposomes are expected to be localized in tissues of the liver and spleen, no or low in vivo radiolabeling of liposomes was observed. A potential approach to overcome this hurdle could be the injection of weakly chelated gallium as the second vector of the pretargeted system (instead of free ^68^Ga), to increase its reach within solid organs.

To further examine the in vivo labeling capabilities of the metal chelation-based pretargeting system based on THP, we set out to explore it using a bone-targeting bisphosphonate tracer (see the Supporting Information for the experimental results). THP-Pamidronate (THP-Pam) (Figure S6A) was chosen for its proven bone-targeting capabilities (Figure S6B–D).^21^ Moreover, a recent study performed by Zeglis et al. has attempted to pretarget a bisphosphonate-based bone tracer with bioorthogonal pretargeting providing an excellent study to contrast between the bioorthogonal approach and our proposed metal pretargeting concept.^5^

As discussed in the previous section, one of the potential explanations for limited observation of pretargeting of liposomes at the second time point of *t* = 24 h could be attributed to the internalization of the liposomes in the soft tissue such as liver and spleen, which is not accessible to ^68^Ga injected later. Therefore, THP-Pam tracer was selected due to its proven biological target (hydroxyapatite in bones) resulting in high accumulation in the joints, thereby potentially retaining THP available for in vivo ^68^Ga chelation. THP-Pam is not expected to be internalized in the soft tissue of organs such as the liver and spleen and should be available for in vivo labeling. The THP-Pam pretargeting experiments (please see the Supporting Information for detailed experimental results of THP-Pam pretargeting experiments) showed the ability of this pretargeting system to react with THP-Pam postclearance and accumulation in the bone tissue. This proves the affinity of THP toward ^68^Ga is high enough to show chelation in vivo in the blood and bone tissue. In the context of the fast blood-clearing THP-Pam, the pretargeting mechanism can be used for pretargeted delivery of both diagnostic and therapeutic radionuclides selectively to target regions such as bones.

## Conclusions

This study explores a new method for ^67^Ga/^68^Ga radiolabeling of liposomal nanomedicines and further evaluates this radiolabeling method for pretargeting *in vivo* using direct ^67^Ga/^68^Ga coordination chemistry. Exploiting the proven gallium/iron-chelating properties of THP, we have developed a method for *in vivo* and *in vitro* radiolabeling of preformed PEGylated liposomes, which can be further used for radiolabeling of different formulations of liposomes with ^68^Ga/^67^Ga with minimal impact on properties. The modification does not interfere with the properties of the PEGylated liposomes used in this study, showing no modification in physical, chemical, and in vivo properties. Our *in vivo* results indicate that radiolabeling utilizing this system is only effective, while the THP-modified liposomes are in the bloodstream and less effective when the liposomes have accumulated in other tissues. Hence, our aim of achieving pretargeted labeling of PEGylated liposomes *in vivo* using this approach was successfully accomplished only at the earlier time point of 5 h post liposomal administration, when most liposomes are still circulating in the blood and not at 24 h post liposomal administration. When using a short-circulating small molecule such as the bone-targeting THP-Pamidronate, moderate pretargeting was observed. This demonstrates that *in vivo* pretargeting using this system is feasible, although not when the targeted component is taken up in organs such as the liver and spleen. Further optimization of the different components may allow for improved outcomes from this strategy.

## Supplementary Material



## Notes

Synthesis scheme, NMR, and high-resolution mass spectrum of THP-phospholipid, RadioTLC characterization of colloid-free ^68^Ga, comparison heat map between different experimental groups of the liposomal pretargeting study, experimental results and discussion of in vivo metal chelation pretargeting of a small molecule, bone-targeting bisphosphonate THP-Pam (PDF)
