# Supplementary material for: Exploring In Vivo Metal Chelation as an Approach for Pretargeted PET Imaging
Source: ACS Omega. 2025 May 7;10(19):19379–93. doi: 10.1021/acsomega.4c10050 (PMC12096208; doi:10.1021/acsomega.4c10050)
Supplement: Supplementary file 1 [file ao4c10050_si_001.pdf]

Supporting information for:

Exploring *in vivo* metal chelation as an approach for pretargeted PET imaging

Aishwarya Mishra[a], George Keeling[a], Jana Kim[a] and Rafael T. M. de Rosales*[a]

[a]School of Biomedical Engineering & Imaging Sciences

King's College London

St Thomas' Hospital

London

SE1 7EH

United Kingdom

* Corresponding author: rafael.torres@kcl.ac.uk

Contents

Section 1. Synthesis and characterisation of THP-phospholipid 3

Section 2. Purification of ^{68}Ga to remove [^{68}Ga]Ga-colloids 6

Section 3. Comparison between different experimental groups of liposomal pretargeting study 7

Section 4. Metal chelation pretargeting of a small molecule: the bone-targeting bisphosphonate THP-Pam 7

Section 1. Synthesis and characterisation of THP-phospholipid

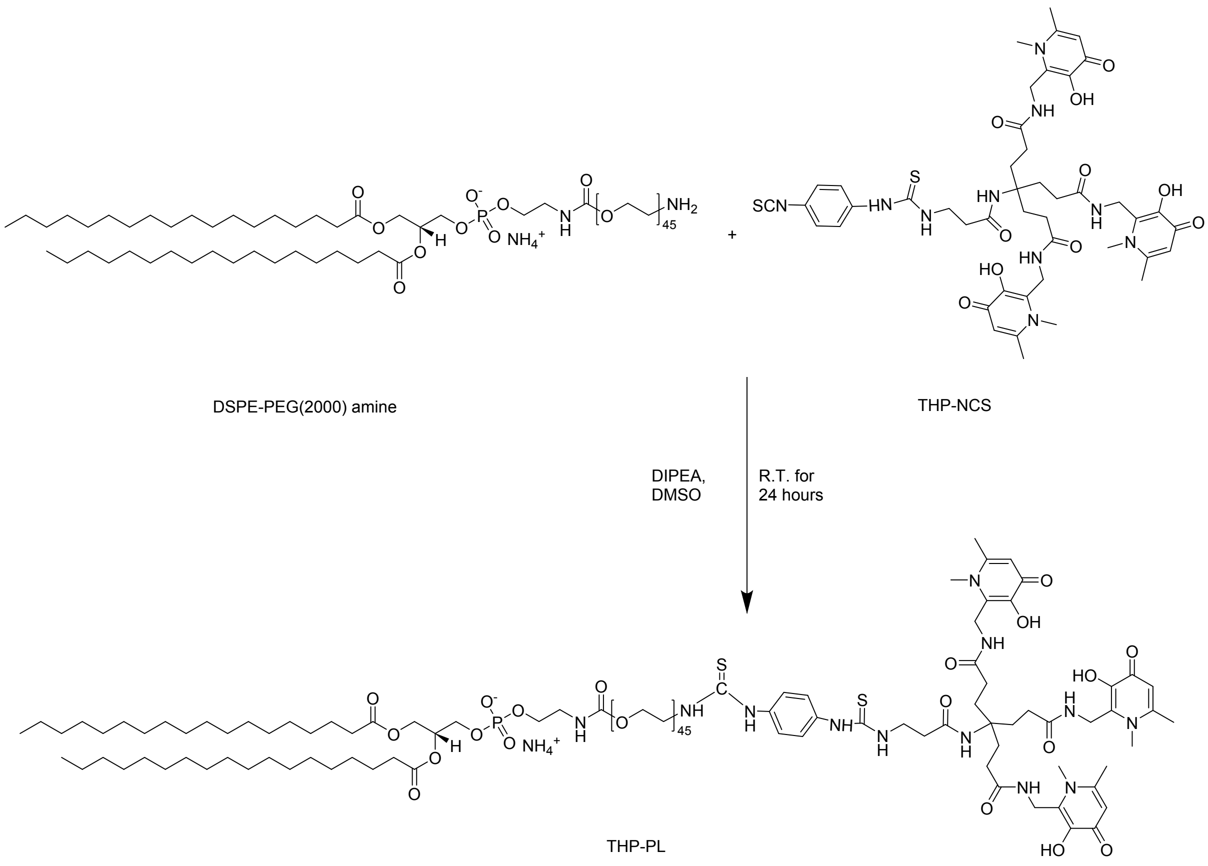

Figure S1. Scheme of the reaction between an Trishydroxypyridinone isothiocyanate and DSPE-PEG2000 (phospholipid) amine

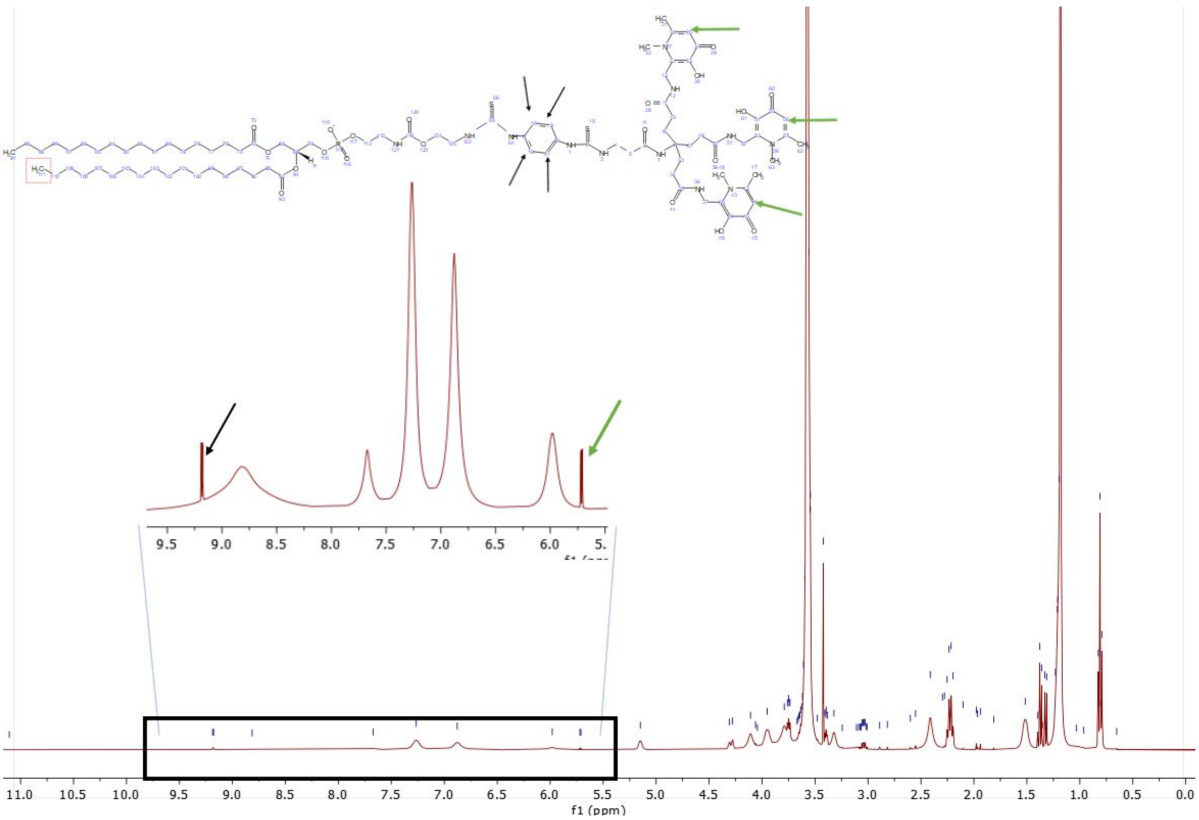

Figure S2. ^{1}H-NMR of THP-PL using 400 MHz NMR in $CDCl_3$ (1024 scans): THP-PL was characterised by the presence of characteristic THP peaks (highlighted using red and black arrows). The purified THP-PL was dissolved in deuterated chloroform and ^{1}H were obtained (H NMR (400 MHz, $CDCl_3$) δ 1H NMR (400 MHz, DMSO) δ 7.45 (s, 1H), 7.27 (s, 1H), 7.19(s, $CHCl_3$), 6.93 (s, 1H), 6.72 (s, 2H), 5.71(s, 3H), 5.15 (s, 1H), 4.32-4.29 (m, 4H), 4.09 (s, 2H), 3.94 (s, 2H), 3.76 (m, 2H), 3.74 (s, 6H), 3.57- 3.55 (m, 180H), 3.42- 3.38 (m, 9H), 3.32(s, 2H), 2.38 (s, 9H), 2.25-2.19 (m, 2H), 2.05 (m, 6H), 1.98 (s, 4H) 1.94 (m, 4H), 1.81 (s, 4H), 1.51 (s, 2H), 1.18 (s, 56H), 0.83- 0.76 (m, 6H)).

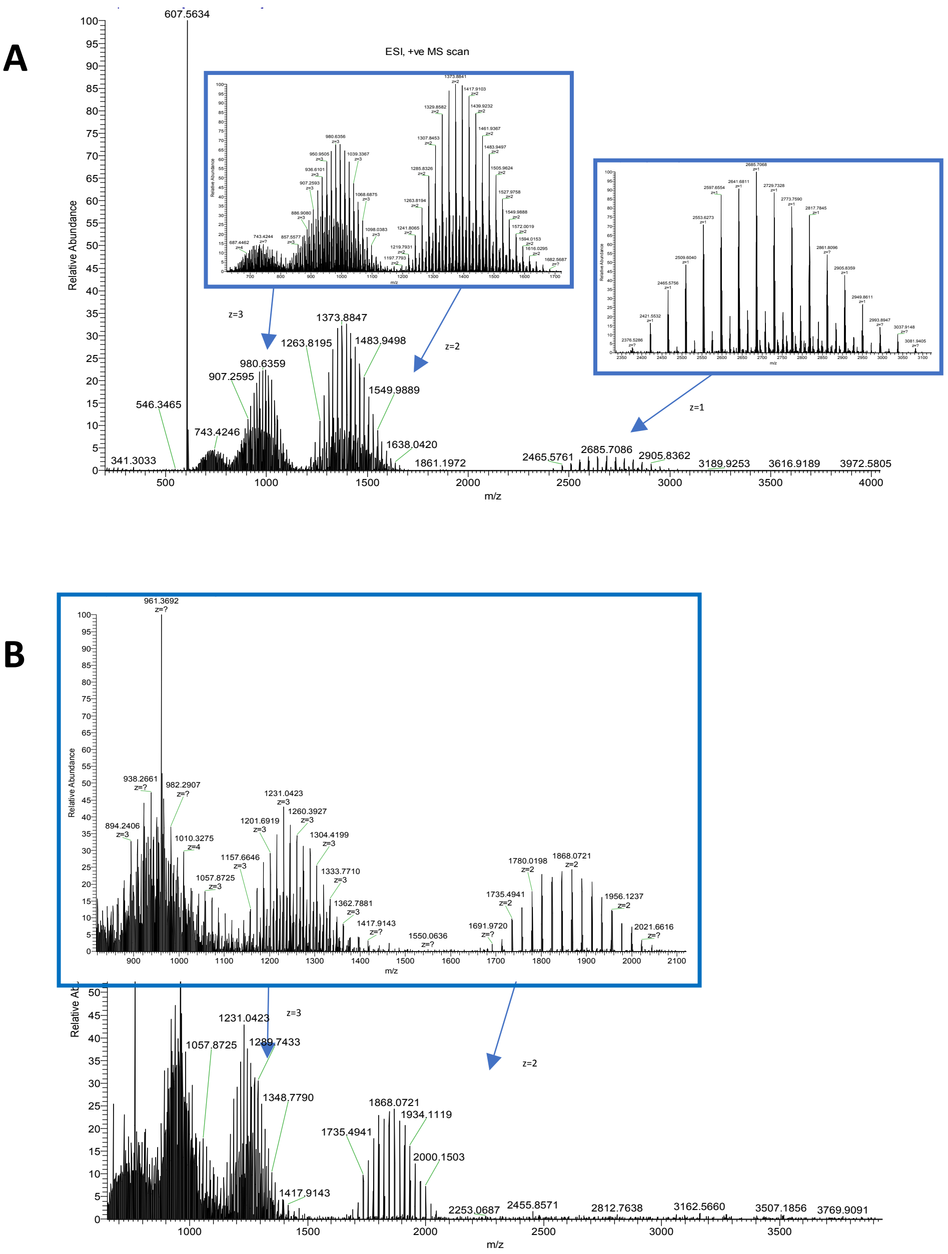

Figure S3. Mass spectra recorded with MALDI-TOF High Resolution Mass spectrometry: The envelope like nature was observed due to characteristic of the mass spectra of PEGylated molecules due to the polydispersity in MW of PEG chains attached to the phospholipid: (A) DSPE-PEG2000-amine: z=1 species 2500-3000 (Exact mass: 2685.7); z=2 species 1250-1650 (Exact mass: 1395.9); z=3 species 800-1100 (Exact mass: 930), (B) THP-PL: z=2 species 1700-2100 (Exact mass: 1866.1); z=3 species 1140-1360 (Exact mass: 1245.1); Z=4 species 840-1060 (Exact mass: 938.5). Base peak in both the spectra was observed at 607.5 due to formation of stable phospholipid fragment on ionization.

Section 2. Purification of ^{68}Ga to remove [^{68}Ga]Ga-colloids

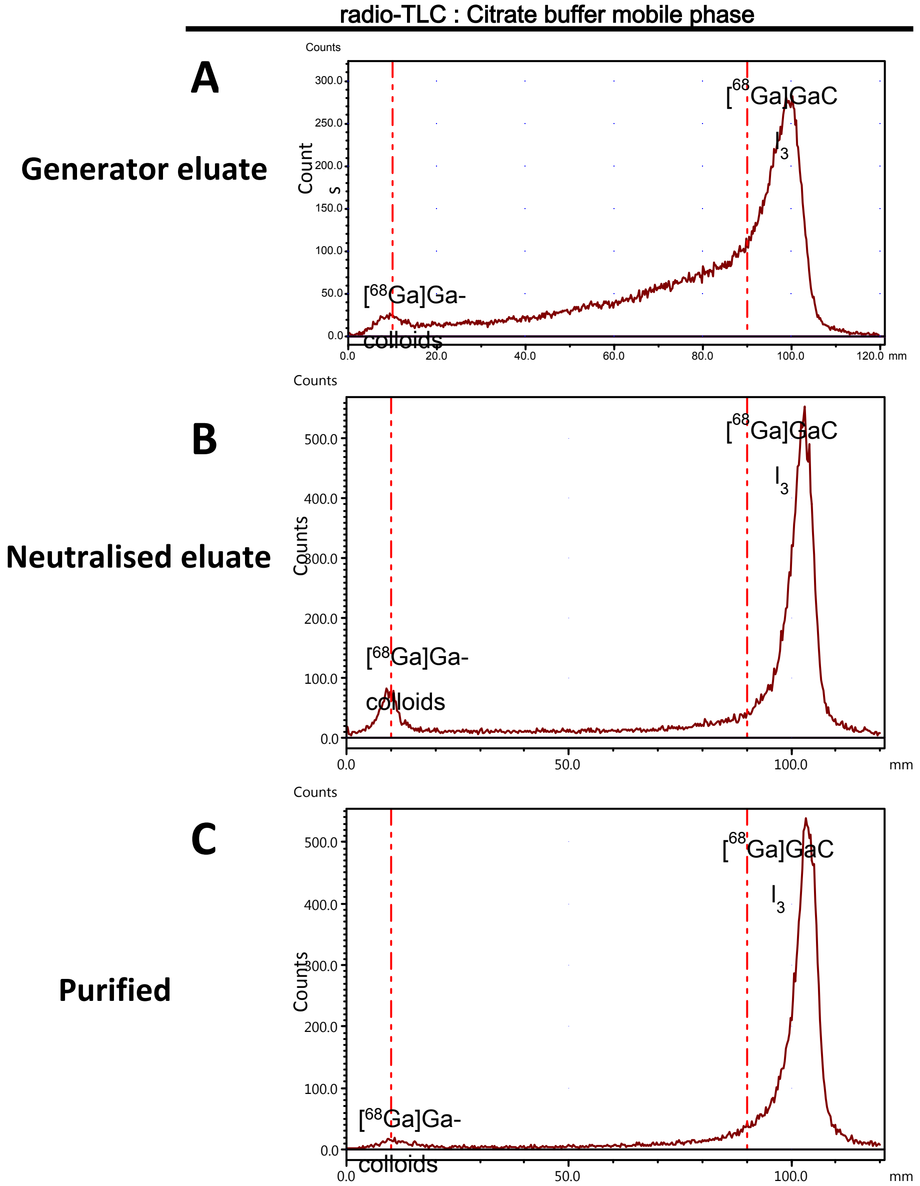

Figure S4. Purification of ^{68}Ga: The colloids generated after neutralisation of gallium generator eluate are removed and the different gallium samples pre and post purification are characterised using ITLC (A) Generator eluate; (B) Neutralised ^{68}Ga pre-purification; and (C) Neutralised ^{68}Ga post-purification

Section 3. Comparison between different experimental groups of liposomal pretargeting study

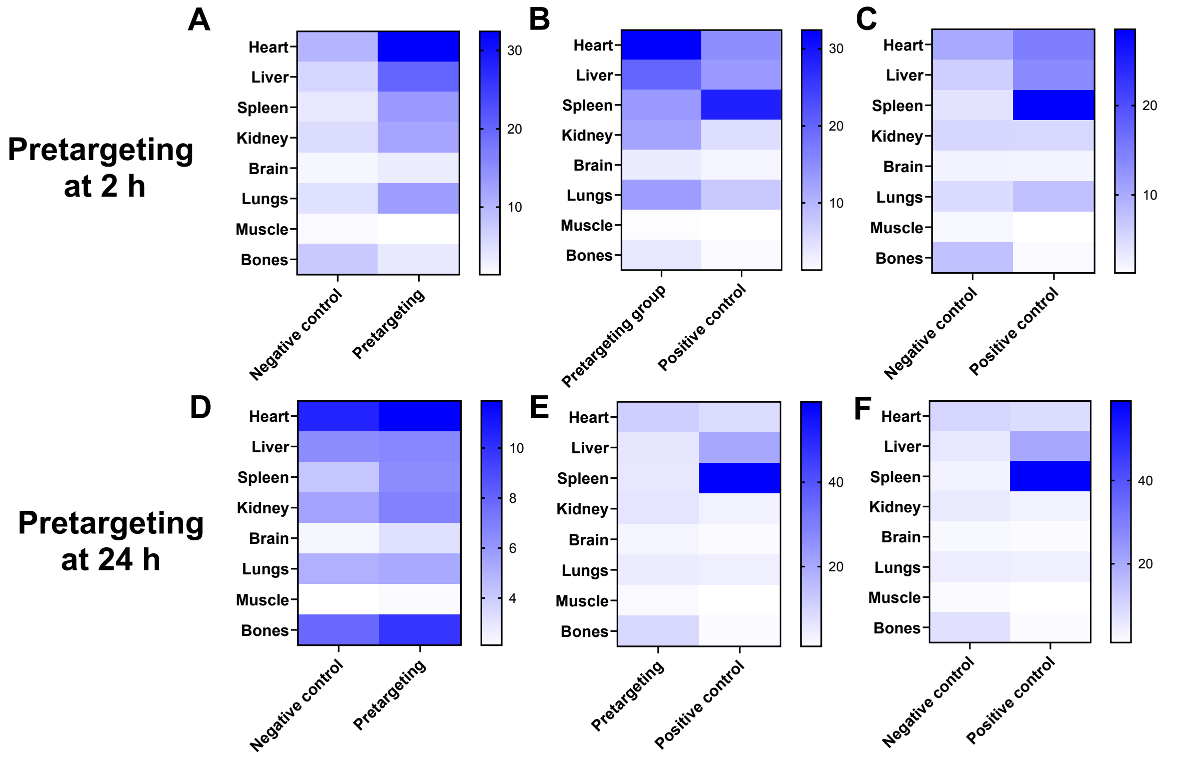

Figure S5. Comparison heat map showing radioactivity uptake in different organs among different experimental groups (in % ID/g): (A) Negative control vs Pretargeting – 2 h; (B) Pretargeting – 2 h vs Positive control- 2 h; (C) Negative control vs Positive control- 2 h; (D) Negative control vs Pretargeting group- 25 h; (E) Pretargeting group- 25 h vs Positive control- 25 h; (F) Negative control vs Positive control- 25 h. A, B, and C heat map are created from image analysis data and D, E, and F heat map are created from biodistribution data.

Section 4. Metal chelation pretargeting of a small molecule: the bone-targeting bisphosphonate THP-Pam

The *in vivo* labelling of THP-Pamidronate was attempted at two time points, t = 2 h and t = 24 h (Fig. S6E-I). The uptake observed was identical at both time points showing high uptake in the urinary bladder, bones specifically joints, and spinal column. The tibia and femur both showed high uptake

values: 10.0 ± 1.3 and 14.0 ± 1.3 %IA/g respectively. Due to the fast clearance of the THP-Pam from the body, low uptake was observed in the liver, large intestine, spleen and kidney and any uptake in these organs might be due to free ^{68}Ga.

The positive control group for bone targeting THP-Pam consisted of a *ex vivo* radiolabelled ^{68}Ga-THP-Pamidronate which was imaged at 1 h p.i. and biodistribution performed at 2 h p.i (Fig. S6B-D). The bone uptake was greater than 20 %IA/g as expected due to its bone-targeting properties and negligible uptake in the rest of the organs due to fast renal clearance.

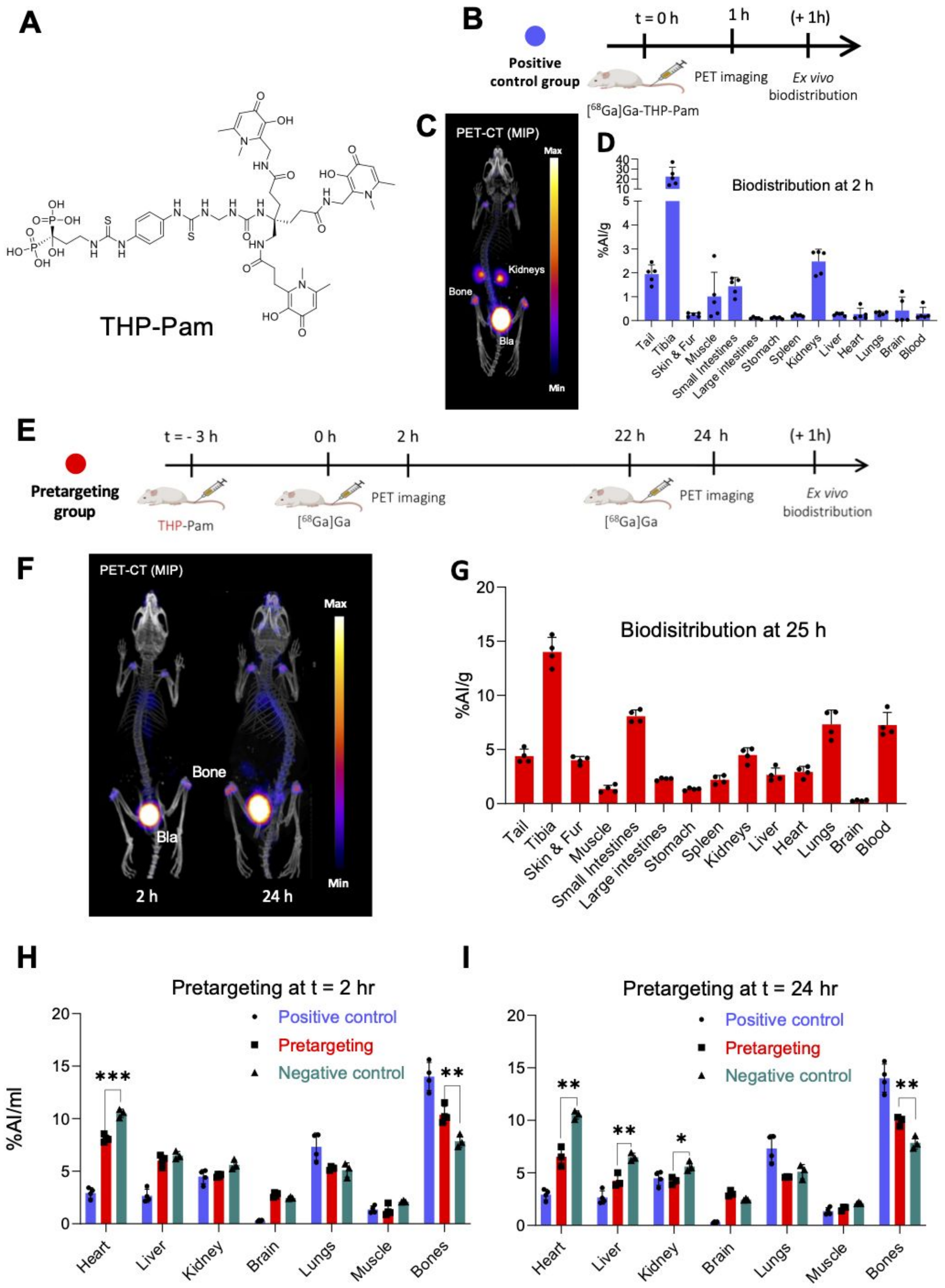

Figure S6. *In vivo* metal chelation pretargeting of small molecule THP-Pam: **(A)** Structure of the bone targeting small molecule THP-Pam with high chelating affinity towards ^{68}Ga; **(B)** Schematic of the positive control *in vivo* experiment of THP-Pam pretargeting; **(C)** *In vivo* PET imaging of ^{68}Ga-THP-Pam at 1 h p.i.; **(D)** Biodistribution performed at 2 h p.i; **(E)** Schematics of the pretargeting group of *in vivo* experiment; **(F)** *In vivo* PET pretargeted imaging of THP-Pam at t = 2 h and t = 24 h; **(G)** Biodistribution at t = 25 h (n=4); **(H, I)** Comparison of image

The success of the metal chelation pretargeting of THP-Pam was determined by the comparison of the bone uptake observed among the different imaging groups (Fig. S6H-I). The PET images from the pretargeting group showed high bone uptake similar to the positive control of ^{68}Ga-THP-Pam. The uptake observed in the bones is higher than the free ^{68}Ga negative control, thereby showing pretargeting. Further quantitative comparison of the biodistribution and quantification of PET images recorded for different experimental groups confirmed these observations of moderate pretargeting (Fig. S6H-I).[33] The directly labelled conventionally targeted THP-Pam showed high bone accumulation (22.5 ± 9 %IA/g) and fast clearance from the blood. Similarly, in the pretargeted THP-Pam group, high uptake was observed in the bones (14 ± 2 %IA/g) and fast clearance of free neutralised ^{68}Ga was observed, with most of the clearance observed via urine. This observation confirmed that the injected THP-Pam could still be traced 24 hours after administration by *in vivo* labelling. The bone uptake trend observed in all analysis methods was Positive control > Pretargeting- 2 h > Pretargeting – 24 h > Negative control.

These results show agreement with observations previously made for pretargeting bisphosphonates using biorthogonal chemistry, including the observation of decreased pretargeting with increased time.[5] Similar to biorthogonal pretargeting, our metal chelation approach provided comparable uptake in the bones and joints for the pretargeting and positive control group. Moreover, as observed in the biorthogonal study by Yazdani *et al.*, decreased blood radioactivity concentration was observed in the pretargeting group compared to free ^{68}Ga (negative control) group (Fig. S6H-I). Thereby confirming the accessibility of the THP-Pam on the targeted bone regions towards free ^{68}Ga for chelation.
